# The EP424R protein of African swine fever virus functions as a 2′-O-methyltransferase and plays an important role in viral replication

**DOI:** 10.1128/mbio.02786-25

**Published:** 2026-03-06

**Authors:** Zixuan Wang, Fenglin Guo, Xueying Wang, Lin Cheng, Yan Liu, Yilin Guo, Sai Niu, Hakimeh Baghaei Daemi, Guiqing Peng

**Affiliations:** 1 State Key Laboratory of Agricultural Microbiology, College of Veterinary Medicine, Huazhong Agricultural University 47895, Wuhan, China; 2 Frontiers Science Center for Animal Breeding and Sustainable Production, Huazhong Agricultural University 47895, Wuhan, China; 3 Hubei Hongshan Laboratory, Huazhong Agricultural University 47895, Wuhan, China; 4 Key Laboratory of Prevention & Control for African Swine Fever and Other Major Pig Diseases, Ministry of Agriculture and Rural Affairs, Wuhan, China; Tsinghua University, Beijing, China

**Keywords:** EP424R protein, African swine fever virus, crystal structure, 2′-O-MTase replication

## Abstract

African swine fever (ASF) is a highly pathogenic infectious disease that poses a serious threat to the pig farming industry worldwide. Currently, there are no effective vaccines and drugs. The 5′ cap structure of messenger RNAs (mRNAs) is a critical modification that is found in all eukaryotic organisms and many viruses. This modification improves mRNA stability by protecting it from cleavage by 5′ to 3′ exonuclease, thus facilitating efficient translation. The African swine fever virus (ASFV) NP868R protein (pNP868R) has 5′-triphosphatase (TPase), guanylyltransferase (GTase), and guanine-N7-methyltransferase (MTase) activities, endowing the viral transcripts with a 7-methylguanosine RNA cap (m^7^GpppN, cap-0). In this study, we found that the *EP424R* gene encodes the 2′-O-ribose methyltransferase (2′-O-MTase) of ASFV, which confers a m^7^GpppNmp-RNA (cap-1) structure on the viral transcripts. Furthermore, we determined the crystal structures of the *EP424R* monomer and the complexes of *EP424R* with *S*-adenosyl-L-methionine (AdoMet, SAM), *S*-adenosyl-L-homocysteine (AdoHcy, SAH), and Sinefungin (SFG). Notably, we identified the key catalytic residues (KDKE) of *EP424R* and clarified its enzymatic mechanism. Additionally, overexpression experiments using the eukaryotic expression plasmid of *EP424R*, along with its mutant plasmid, and specific small interfering RNA (siRNA) interference assays in wild boar lung (WSL) cells demonstrated that this gene is critical for replication of the ASFV. Furthermore, this role is partially mediated by its methyltransferase activity. In summary, our structural and biochemical studies elucidated the process of ASFV mRNA capping, and subsequent virological investigations confirmed the significance of this gene in ASFV replication, highlighting its potential as an antiviral target.

**IMPORTANCE:**

2′-O-ribose methyltransferases (2′-O-MTases) play a vital role in cellular processes of eukaryotes and in the replication of many viruses. However, these enzymes in the African swine fever virus (ASFV) remain uncharacterized. Our study revealed that the EP424R protein (pEP424R) of ASFV exhibits 2′-O-MTase activity during viral replication and is crucial for ASFV replication. Notably, this function is partially dependent on its methyltransferase activity. These findings increase our understanding of the transcriptional capping mechanism of ASFV and provide new insights for developing antiviral drugs targeting African swine fever (ASF).

## INTRODUCTION

African swine fever (ASF) is an acute viral hemorrhagic disease that is caused by African swine fever virus (ASFV), and it affects domestic pigs and wild boars, with fatality rates close to 100% (1, 2). ASF was first identified in Kenya in 1921 and rapidly spread to other countries (3, 4). The virus can be transmitted by direct contact with infected pigs or their products and by bites from soft ticks (2, 5). The disease spreads rapidly in pigs (6). The World Organization for Animal Health has listed ASF as a notifiable animal disease, and China has classified it as a class I animal disease (7, 8). Currently, there are no effective vaccines or drugs against the disease, and it can be prevented and controlled only through culling. Therefore, ASF has severely affected the healthy development of the pig industry and has caused substantial economic losses (9).

In eukaryotic cells, the 5′ end capping system is essential for stabilizing messenger RNA (mRNA), promoting translation initiation, and regulating nucleocytoplasmic transport. This system enhances the efficiency and accuracy of gene expression (10, 11). Furthermore, the capping modification of host mRNA serves as a “self” marker, enabling cells to recognize exogenous RNAs (including viral transcripts) that lack such capping as “non-self,” thereby triggering innate immune responses (12, 13). However, many viruses that replicate within the cytoplasm of eukaryotic cells have evolved mechanisms to cap their own mRNAs, allowing them to evade recognition by the innate immune system and promoting viral proliferation (11, 14–16). The mRNA capping process involves four enzymatic reactions.

The γ-phosphate at the 5′ end of the primary transcript is specifically hydrolyzed by triphosphatase (TPase) to generate 5′-diphosphate RNA (ppNp-RNA). Then, the ppNp-RNA is covered with GMP by guanylyltransferase (GTase), thereby forming GpppNp-RNA. Next, (guanine-N7)-methyltransferase (N7-MTase) methylates the GpppNp-RNA to form m^7^GpppNp-RNA (cap-0). Finally, 2′-O-ribose methyltransferase (2′-O-MTase) further methylates the first nucleotide from the 3′ to the triphosphate bridge to produce m^7^GpppNmp-RNA (cap-1) and continues to methylate the second nucleotide to form m^7^GpppNmpNmp-RNA (cap-2) (15, 17). However, it’s important to note that the cap-2 structure is not present on all mRNAs (18, 19). These four enzymes are encoded by different genes in eukaryotic cells and viruses (14, 20–22). In large DNA viruses, the baculovirus protein LEF-4 has bifunctional TPase-GTase activity, but it does not contain the N7-MTase and 2′-O-MTase domains (22); the RNA capping mechanism of vaccinia virus (VACV) consists of the D1 subunit, the stimulatory D12 subunit, and VP39; the enzymatic activities of the N-terminal TPase domain, the central GTase domain, and the C-terminal MTase domain of the D1 subunit have been well characterized; and VP39 is a bifunctional protein that acts as a 2′-O-MTase and a poly(A) polymerase stimulatory factor (14, 20).

Many members of the nucleocytoplasmic large DNA viruses (NCLDVs) undergo transcription and post-transcriptional modifications in the cytoplasm (23), which require virus-encoded proteins to produce mature mRNA from the viral genome. ASFV is the only known arbovirus with a mature virion genome that is 170–190 kb in length and has 150 to 167 open reading frames, encoding viral proteins involved in genome replication, transcription and translation, virion assembly, and immune escape (2, 5, 24). In the previous studies, ASFV was shown to encode core subunits and general transcription factors to form an RNA polymerase (RNAP) -like transcription apparatus that drives the initiation, elongation, and termination of viral gene transcription (25, 26). The structure of ASFV RNAP (vRNAP) is composed of eight core subunits (vRPB1, vRPB2, vRPB3-11, vRPB5, vRPB6, vRPB7, vRPB9, and vRPB10) (27). In addition, an additional subunit called *M1249L* is integrated into the RNAP core complex in a specific manner to regulate RNAP. The pM1249L domain interacts with almost all the core subunits of RNAP, thus forming a caged structure to increase the stability of the complex (23, 28). ASFV DNA is transcribed into pre-mRNA via vRNAP and transcription factors (25). Subsequently, the pre-mRNA of ASFV forms a cap-0 structure under the action of pNP868R (11). However, no study has yet determined which gene is responsible for the formation of the cap-1 structure. The capped mRNA forms a poly(A) tail at the 3′ end under the action of the poly (A) polymerase C475L (29). Finally, it undergoes exon splicing to become mature mRNA (30).

The EP424R protein (pEP424R) is a structural protein of ASFV that is encoded by the *EP424R* gene (31). We speculate that pEP424R participates in the process of viral mRNA capping by functioning as a 2′-O-MTase. Currently, the predicted function of this gene is coding for an FTSJ-like RNA methyltransferase (32, 33), but its specific role in the ASFV life cycle has not been experimentally validated. In this study, we systematically characterized the function of the target protein through comprehensive structural and biochemical analyses, ensuring the validity of our findings. Moreover, a series of virological experiments further confirmed that this protein plays an indispensable and crucial role in the ASFV replication process. Our research provides not only structurally informative data of significant reference value but also a solid foundation for elucidating ASFV transcription and post-transcriptional processing. Furthermore, our findings suggest potential candidate targets for the screening and design of novel anti-ASF drugs.

## RESULTS

### Overall structure of ASFV pEP424R

**Fig 1:**
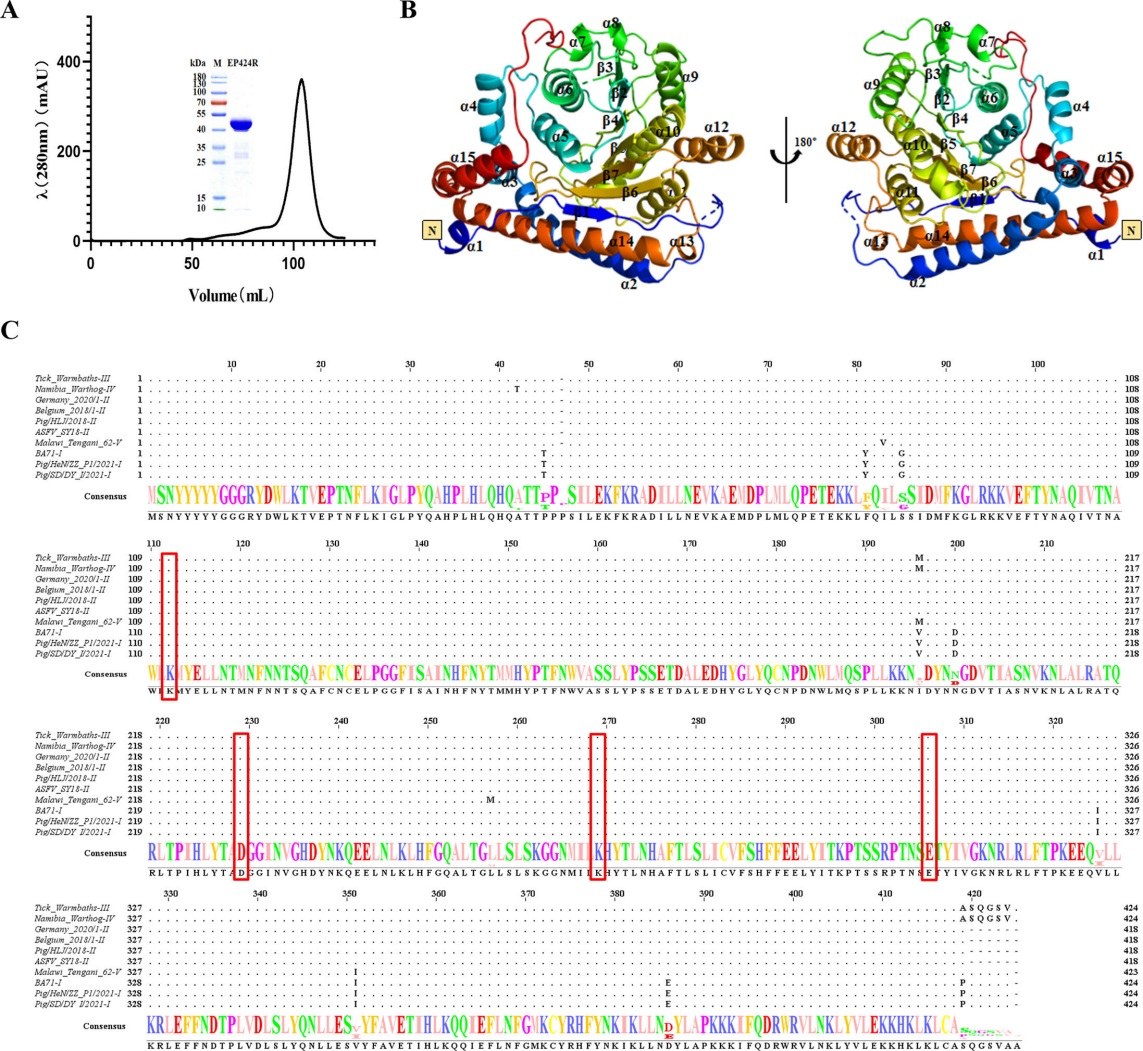
Stereo view of the ASFV pEP424R structure. (**A**) Analytical gel filtration of pEP424R. The 280 nm absorbance curve from the Superdex 200 column and the SDS-PAGE migration profile of the pooled sample are shown. (**B**) Crystal structure of monomeric ASFV pEP424R. The structure of pEP424R is shown as a cartoon in colors ranging from blue in the N-terminal region to red in the C-terminal region. (**C**) Sequence alignment of ASFV pEP424R in different genotypes. The following sequences from GenBank were used for sequence alignment: ASFV-SY18 (genotype II), accession number MH766894.1; Tick_Warmbaths (genotype III), AY261365; Namibia_Warthog (genotype IV), AY261366; Pig/HLJ/2018 (genotype II), MK333180; Belgium_2018/1 (genotype II), LR536725; Germany_2020/1 (genotype II), LR899193; Malawi_Tengani_62 (genotype V), AY261364; BA71V (adaptive strain), NC_001659.2; Pig/HeN/ZZ_P1/2021 (genotype I), MZ945536; and Pig/SD/DY_I/2021 (genotype I), MZ945537. Dots represent identical aa, and capital letters represent the different aa. Possible key enzyme active sites are highlighted with red boxes and selected them in the subsequent mutagenesis studies.

The structure of native ASFV pEP424R was solved by molecular replacement, using the structural model of pEP424R generated by AlphaFold 2 (34) as a search model. pEP424R was crystallized in the P1211 space group, and the final model was refined at a 2.79 Å resolution to *R*_work_ and *R*_free_ values of 20.25% and 25.99%, respectively (Table 1). Two protein molecules form a homodimer by crystal packing in a symmetric unit (Fig. S1A). However, the Ni affinity-purified pEP424R was eluted through a HiLoad 16/600 Superdex 200 pg column with an elution volume of approximately 100 mL, and SDS-PAGE revealed that the molecular weight of the pEP424R monomer was 50 kDa, indicating that pEP424R is a monomer in solution (Fig. 1A).

**TABLE 1:** X-ray data collection and refinement statistics^*a*^

	ASFV MTase	ASFV MTase-SFG	ASFV MTase-SAM	ASFV MTase-SAH
Data collection statistics				
X-ray source	SSRF BL10U2	SSRF BL02U1	SSRF BL02U1	SSRF BL19U1
Wavelength (Å)	0.97918	0.97918	0.97918	0.97923
Space group	P1211	P1211	P1211	P1211
Cell constants	a = 80.254 Å b = 57.939 Å c = 102.143 Å α = 90.00° β = 112.162° γ = 90.00°	a = 80.211 Å b = 57.777 Å c = 101.912 Å α = 90.00° β = 112.048° γ = 90.00°	a = 79.5957 Å b = 57.7227 Å c = 101.429 Å α = 90.00° β = 111.443° γ = 90.00°	a = 162.39 Å b = 162.39 Å c = 162.39 Å α = 90.00° β = 90.00° γ = 90.00°
Resolution (Å)	50.00–2.79 (2.89–2.79)	19.49–2.88 (2.983–2.79)	50.07–2.59 (2.683–2.59)	48.42–2.422 (2.49–2.42)
Unique reflections	21,806 (2,194)	18,287 (1,845)	26,688 (2,590)	31,642 (2,304)
Completeness (%)	99.17 (99.73)	91.87 (94.66)	98.72 (97.95)	99.20 (97.18)
Mean I/sigma (I)	5.66 (2.84)	18.65 (9.00)	6.70 (2.95)	10.05 (4.94)
Redundancy	2.8 (2.8)	5.1 (5.2)	6.1 (5.0)	6.63 (5.76)
*R_merge_* (%)^*b*^	16.66 (41.62)	7.073 (15.11)	26.23 (85.33)	19.16 (20.03)
*R_measure_* (%)^*c*^	20.6 (51.21)	7.86 (16.72)	28.83 (96.11)	20.87 (21.93)
*R_pim_* (%)^*c*^	11.95 (29.44)	3.336 (6.996)	11.74 (43.15)	8.11 (8.77)
CC_1/2_ (%)	95.7 (73.5)	99.5 (98.4)	92.5 (42.4)	86.33 (64.02)
Refinement statistics				
Resolution range (Å)	50.00–2.79	19.49–2.88	50.07–2.59	48.54–2.42
*R_work_*/*R_free_* (%)^*d*^	20.23/26.20	18.12/18.82	24.16/27.67	20.46/21.23
Protein atoms	6,528	6,426	6,467	6,482
Ligand atoms	0	54	54	26
Water atoms	221	193	161	231
R.m.s. deviations				
Bond lengths (Å)	0.004	0.003	0.011	0.011
Bond angles (°)	0.69	0.68	1.56	1.41
Ramachandran plot (%)				
Favored	96.14	96.03	95.31	95.78
Allowed	3.86	3.57	4.43	4.22
Outliers	0	0.40	0.26	0
PDB code	9W69	9W7G	9W7F	9IQ4

^*a*^ Values in parentheses represent the highest resolution shell.

^*b*^ *R*_*merge*_ = *Σ*_*hkl*_*Σ*_*i*_|*I*(*hkl*)_i_−<*I*(*hkl*)>|/*Σ*_*hkl*_*Σ*_i_*I*(*hkl*)_i_, where *I*(*hkl*) is the intensity of reflection *hkl* and its symmetry equivalents, and <*I*(*hkl*)> is the average intensity over all equivalent reflections.

^*c*^ *R*_*measure*_: multiplicity-weighted *R*_*merge*_; *R*_*pim*_: precision-indicating *R*_*merge*_.

^*d*^ *R* =* Σ*||Fo| − |Fc||/*Σ*|Fo| · |Fo| and |Fc| are amplitudes of the observed and calculated structure factors, respectively. *R*_*work*_ is the *R* value for reflections used in the refinement, whereas *R*_*free*_ is the *R *value for 5% of the reflections, which are selected in thin shells and are not included in the refinement.

The crystal structure of pEP424R contains fifteen helices and a seven-strand β-sheet, which has a classical Rossmann-fold structure and exhibits typical folding features of the class I MTase family. The seven-strand β-sheet (described as β3, β2, β4, β5, β7, and β1) is located in the center of the structure. The plane composed of the seven-strand β-sheet is surrounded by three helices on each side (α5, α6, and α7 on one side and α9, α10, and α11 on the other side) (Fig. 1B).

The amino acid (aa) sequences of pEP424R from 10 ASFV isolates were analyzed by multiple sequence alignment by Clustal W (https://www.genome.jp/tools-bin/clustalw) and ESPript 3.0 (https://espript.ibcp.fr/ESPript/ESPript/index.php). We found that the aa sequence identities varied from 96% to 100% (Fig. 1C), indicating high-level conservation of pEP424R among various ASFV isolates.

### ASFV pEP424R displays 2′-O-MTase activity

**Fig 2:**
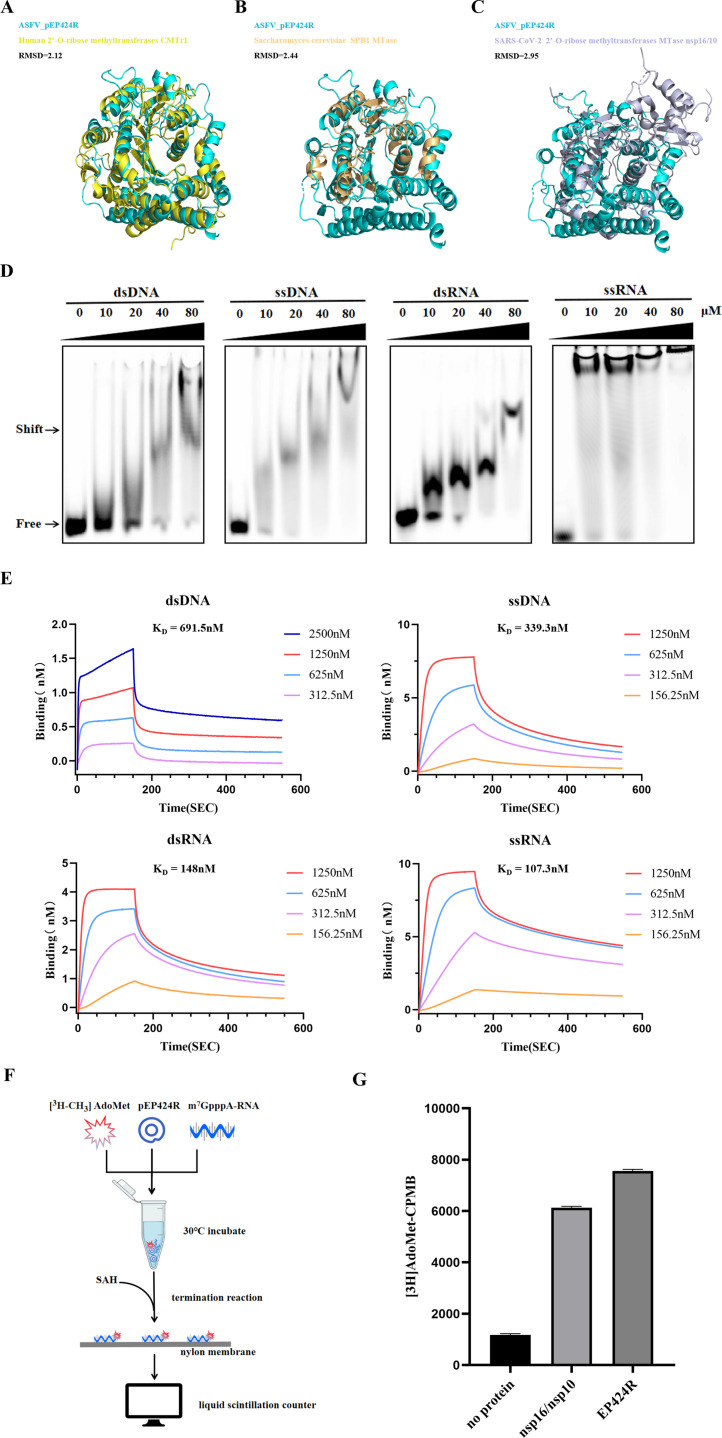
Structural alignment and functional validation of pEP424R. (**A**) Superimposition of the human 2′-O-MTase domain CMTE1 (pdb entry 4N48, yellow) on ASFV pEP424R (cyan). (**B**) Superimposition of the *Saccharomyces cerevisiae* SPB1 MTase (pdb entry 7R7O, light orange) on ASFV pEP424R (cyan). (**C**) Superimposition of the SARS-CoV-2 2′-O-MTase nsp16/10 (pdb entry 7L6R, light blue) on ASFV pEP424R (cyan). (**D**) Electrophoretic mobility shift assay (EMSA) was performed to analyze the binding between ASFV pEP424R and nucleic acids. Various concentrations of pEP424R (0, 10, 20, 40, and 80 µM) were assayed with 1 µM 5′-Cy5-labeled nucleic acid fragments. The arrow indicates the mobility shift of Cy5-labeled oligos. (**E**) Biolayer interferometry (BLI) was performed to analyze the binding kinetics between pEP424R and nucleic acids. One micromolar biotin-labeled nucleic acid was immobilized on streptavidin (SA) biosensors and incubated with twofold serially diluted pEP424R. (**F**) Flow chart of the radioisotope labeling experiment. (**G**) The MTase activity of pEP424R with [^3^H–CH_3_] AdoMet and m7GpppA-capped RNA substrates was used as indicated. Data points are presented as the mean values ± standard deviations (*n* = 3).

The biological role of ASFV pEP424R has not been determined. The PDBefold server (https://www.ebi.ac.uk/msd-srv/ssm/) was used to search for the pEP424R structure to identify structural homologs within the RCSB PDB (35). Significant structural similarity was detected between pEP424R and the MTases from different species despite ∼20% aa sequence identity (Fig. S2A). The three closest structural homologs are the MTase domain of the 2′-O-ribose MTase CMTR1 from humans (Fig. 2A; PDB ID 4N48), the rRNA MTase SPB1 from *Saccharomyces cerevisiae* (Fig. 2B; PDB ID 7R7O), and the severe acute respiratory syndrome coronavirus 2 (SARS-CoV-2) 2′-O-ribose MTase nsp16/10 (Fig. 2C; PDB ID 7L6R).

The 3D structure of pEP424R is most similar to that of the human-encoded 2′-O-ribose MTase CMTR1, with an RMSD value of 2.12. CMTR1 catalyzes the methylation of the 2′-O-ribose of the first nucleotide of mRNA transcripts, which can protect the transcripts from exonuclease degradation and improve the stability and translation efficiency of mRNA (36, 37).

To confirm whether ASFV pEP424R has the same capping function as CMTR1 does, we first evaluated the nucleic acid binding preference of pEP424R by an electrophoretic mobility shift assay (EMSA) and biolayer interferometry (BLI). The EMSA results revealed that pEP424R strongly bound to the various oligonucleotides tested, and its mobility in the gel changed in a dose-dependent manner with the concentration of pEP424R (Fig. 2D). Similar results were obtained with the BLI assay. The *K_D_* values for ssRNA, dsRNA, ssDNA, and dsDNA were 107.3, 148, 339.3, and 691.5 nM, respectively (Fig. 2E). Furthermore, the affinity of pEP424R for the ssRNA fragments was significantly greater than that for the ssDNA, dsDNA, and dsRNA fragments, which was consistent with our expectations. Next, we conducted an enzyme activity experiment with pEP424R *in vitro*. The 2′-O-MTase activity of pEP424R was detected by labeling the transfer from [^3^H–CH_3_] AdoMet to m7GpppA-capped RNA (Fig. 2F). The nsp16/nsp10 of feline infectious peritonitis virus (FIPV) was used as the positive control, and no protein was added as the negative control. The results showed that pEP424R could transfer the methyl groups on AdoMet to m7GpppA-RNA (Fig. 2G).

### ASFV 2′-O-MTase shares a conserved key enzymatic active site with other viruses

Through the above biochemical experiments, we determined that pEP424R possesses 2′-O-MTase enzymatic activity. Therefore, we also resolved the structures of the pEP424R complex with S-adenosyl-L-methionine (AdoMet, SAM), which is the methyl donor of universal methyltransferase, and Sinefungin (SFG), which is a broad MTase inhibitor.

**Fig 3:**
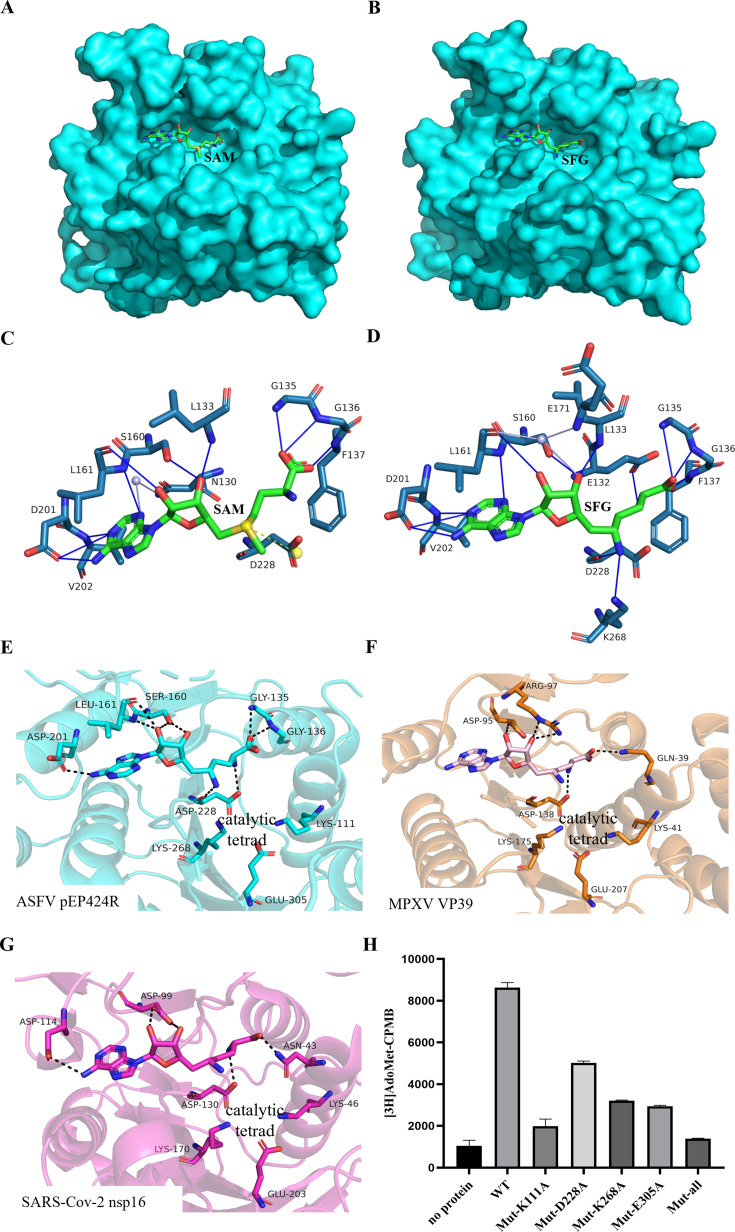
Determination of the key enzymatic active site in ASFV pEP424R. (**A**) Structure of pEP424R in complex with SAM. pEP424R (cyan) is shown in surface representation, while SAM is shown in stick representation and colored according to its elements: carbon, green; nitrogen, blue; oxygen, red. (**B**) SFG is bound at the SAM binding site. (**C**) Detailed view of SAM or (**D**) SFG in the binding site. The SAM, SFG, and side chains of the selected pEP424R amino acid residues are shown as sticks. The carbon atoms of the selected pEP424R amino acid residues are colored according to the protein assignment, the carbon atoms of SAM and SFG are shown in green, and other elements are colored as usual. Selected hydrogen bonds involved in the EP424R-SAM or SFG interaction are presented as solid blue lines. Water molecules are shown as light blue spheres. Water bridges are represented by solid light blue lines, and salt bridges are represented by dashed yellow lines. (**E–G**) Structural alignment of the MTase active sites of ASFV pEP424R with those of the monkeypox virus VP39 and SARS-CoV-2 nsp16. Crystal structures of monkeypox virus (MPXV) VP39 (F, pdb entry 8B07) and SARS-CoV-2 nsp16/nsp10 (G, pdb entry 6YZ1) were used for alignment. Protein backbones are shown in cartoon representation and depicted in cyan (ASFV pEP424R), orange (monkeypox virus VP39), and light magenta (SARS-CoV-2 nsp16). SFG and the side chains of selected residues are shown in stick representation with carbon atoms colored according to the protein assignment and other elements colored as in panel A. (**H**) *In vitro* MTase activities of the pRP424R variants. The proteins were assayed for MTase activity by label transfer from [^3^H–CH_3_] AdoMet to m7GpppA-capped RNA as described in the Materials and Methods. Data points are presented as the mean values ± standard deviations (*n* = 3).

The structures of the two complexes could be resolved upon molecular replacement in the central pocket of the pEP424R MTase, and SFG occupied the SAM binding pocket (Fig. 3A and B). The adenine moiety of SAM formed hydrogen bond interactions with residues V202, D201, L161, and S160 (Fig. 3C). The ribose and amino acid moieties were also involved in hydrogen bonding: the ribose ring directly bound to L161, S160, and L133 through its hydroxyl group, while the amino acid moiety formed hydrogen bonds with G135, G136, and F137 (Fig. 3C), similar to SFG (Fig. 3D). In contrast, amino acids of SFG also formed hydrogen bonds with E132 (Fig. 3D). In addition, the S atom of SAM formed a salt bridge with the side chain of residue D228 (Fig. 3C), while the amino group of SFG formed a hydrogen bond interaction with residues D228 and K268 (Fig. 3D). The adenine N3 of SAM formed a water bridge with the side chain of residue N130 through water molecules (Fig. 3C), while the ribose ring hydroxyl group of SFG formed a water bridge with the side chains of residues L161 and E171 (Fig. 3D). The similar interaction forces between the two small molecules and the protein also provided a structural basis for SFG as an inhibitor of methyl transfer.

Next, we compared the catalytic site of pEP424R with that of monkeypox virus (MPXV), which also belongs to the NCLDV family, and the 2′-O-MTase of the unrelated important virus SARS-CoV-2. The resemblance of the SAM binding sites was notable in both cases. SFG was present in virtually the same conformation in ASFV pEP424R, MPXV VP39, and SARS-CoV-2 nsp16 (Fig. 3E through G). The catalytic tetrad (LYS111, ASP228, LYS268, and GLU305 for ASFV) was perfectly conserved among these different viruses, including the conformation of these residues. These four amino acids of pEP424R were also perfectly conformed in different genotype strains (Fig. 1B). Therefore, to verify whether these four residues constitute the key catalytic sites of pEP424R, we constructed a series of alanine-scanning mutants. This included four single-point mutants and one quadruple mutant. All corresponding proteins were expressed and purified. The enzyme activity test was subsequently conducted again using radioactive isotope biochemical experiments. The results indicated that the enzymatic activities of the five mutant proteins decreased significantly compared with those of the wild-type proteins, and the enzymatic activities of the protein with all four amino acids mutated decreased to the lowest level (Fig. 3H). The ssRNA-binding affinity of the quadruple mutant (EP424R^4A^) was also assessed. BLI assay revealed a significant decrease in binding affinity relative to the wild-type protein, demonstrating that these residues are critical for ssRNA binding. (Fig. 2E; Fig. S3A).

Therefore, LYS111, ASP228, LYS268, and GLU305 are the key enzymatic active sites of ASFV 2′-O-MTase.

### Mechanism of action of the key enzyme active sites of pEP424R

**Fig 4:**
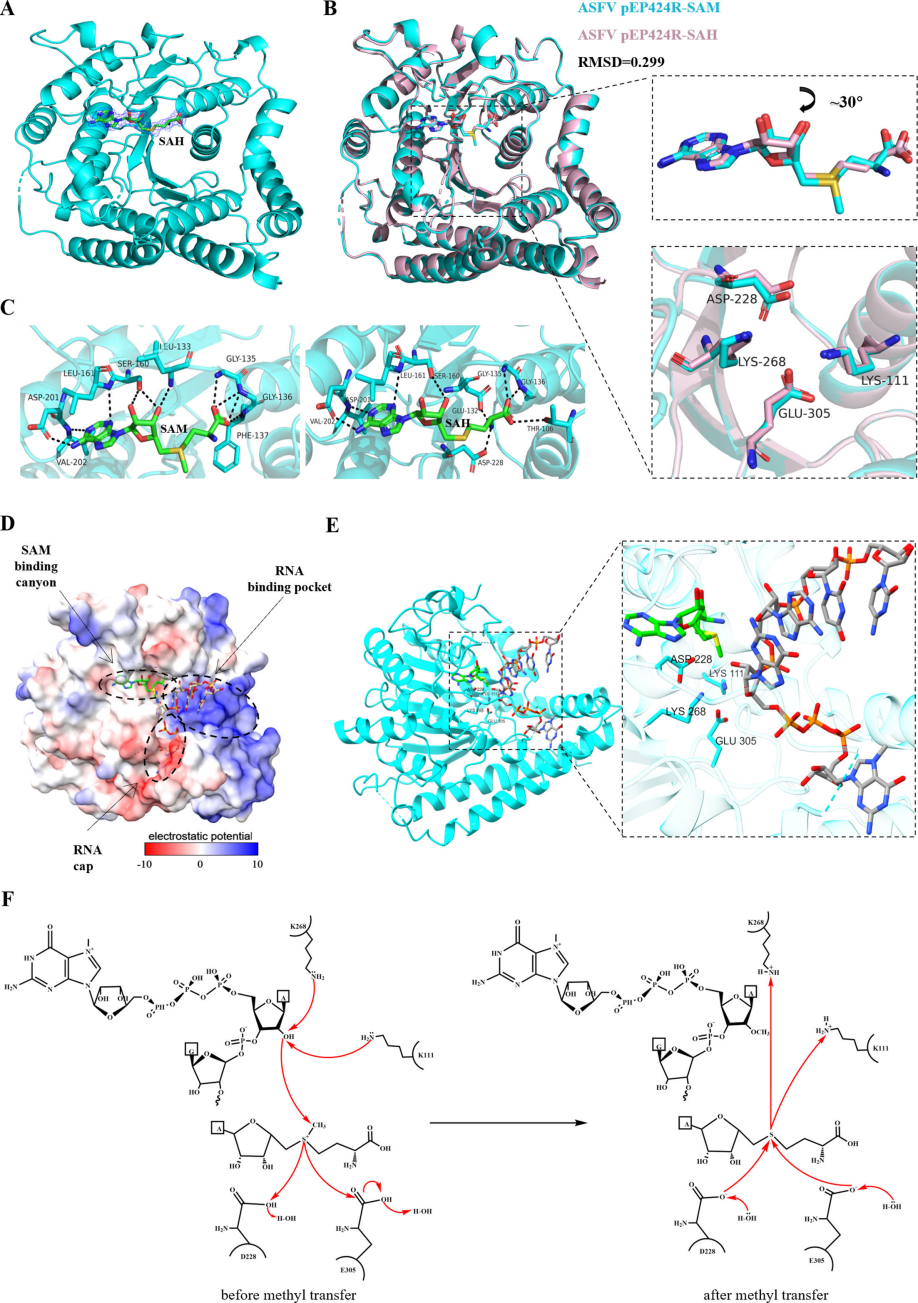
Mechanism of action of the pEP424R key enzyme active site. (**A**) Overall fold change in pEP424R in complex with SAH. The protein backbone is shown in cartoon representation and depicted in cyan, while SFG is shown in stick representation and colored according to its elements: carbon, green; nitrogen, blue; oxygen, red. The Fo–Fc omit map contoured at 1σ is shown around SAH, and the map color is blue. (**B**) Superimposition of pEP424R-SAH (light pink) on pEP424R-SAM (cyan). SAM, SAH, and the side chains of selected residues are shown in stick representation with carbon atoms colored according to the protein assignment and other elements colored as in Fig. 3A. (**C**) Detailed view of SAM or SAH in the binding site. The SAM, SAH, and side chains of the selected pEP424R amino acid residues are shown as sticks. The carbon atoms of the selected pEP424R amino acid residues are colored according to the protein assignment, the carbon atoms of SAM and SAH are shown in green, and other elements are colored as usual. Selected hydrogen bonds involved in the EP424R-SAM or SFG interaction are presented as dashed black lines. (**D**) Model of RNA recognition by ASFV pEP424R; the surface of pEP424R is colored according to the electrostatic surface potential. m7GpppG-capped RNA was modeled by structural alignment using the crystal structure of the human 2′-O-MTase CMTR1 in complex with SAM and m7GpppG-capped RNA (pdb entry 4N49) as a template. SAM and m7GpppG-capped RNA are shown as sticks. The carbon atoms of SAM are shown in green, the carbon atoms of m7GpppG-capped RNA are shown in gray, and the other elements are colored as usual. (**E**) Cartoon of the ASFV pEP424R recognition RNA model. The presentation form and elemental coloring of SAM and m7GpppG-capped RNA are consistent with those in panel D. The selected pEP424R amino acid residues are shown in the stick representation with carbon atoms colored according to the protein assignment; the phosphorus element is shown in orange, and the other elements are colored as usual. (**F**) The process by which the four key enzyme active sites of pEP424R transfer methyl groups.

Next, we aimed to explore the specific mechanism of action of the four key active sites of pEP424R. To achieve this, the structure of the pEP424R complex with *S*-adenosyl-L-homocysteine (SAH) after methyl transfer was resolved. The electron density of SAH was immediately visible upon molecular replacement in the central pocket of the pEP424R MTase (Fig. 4A). We superimposed the structures of the pEP424R-SAM and pEP424R-SAH complexes and found that the overall protein structure did not change significantly after the reaction, with an RMSD value of only 0.299 (Fig. 4B). We further superimposed the active pockets and found that the conformations of the amino acids did not change significantly, except for a few amino acids that experienced relative displacements (Fig. S3). Additionally, we observed that the conformations of the four key enzymatic sites, LYS111 and ASP228, remained completely unchanged, while the side chain conformations of LYS268 and GLU305 were altered, which might be due to electron transfer during the reaction; the pentose sugar of the small molecule rotated by 30°, and its methionine part changed from the S conformation to the R conformation (Fig. 4B). These might also explain the slight differences in the interaction forces between the two small molecules and the protein (Fig. 4C).

On the basis of the previously solved crystal structure of the human 2′-O-MTase CMTR1 in complex with RNA (37), we constructed a model of a ternary complex SAH/RNA/pEP424R complex to illustrate the molecular mechanism through which pEP424R transfers methyl groups (Fig. 4D). Therefore, we describe the process by which pEP424R transfers methyl groups: the residues LYS111 and LYS268 of pEP424R deprotonate the hydroxyl group on the 2′-O-ribose, and the negatively charged oxygen ion attacks the sulfur methyl carbon on SAM; the methyl carbon breaks away from the S atom and transfers to the 2′-O-ribose, forming a methyl oxygen, thereby completing the capping of the cap-1 structure. In addition, the carboxyl groups of the active center residues ASP228 and GLU305 carry negative charges under physiological conditions, which helps maintain the charge balance in the active center after the hydroxyl deprotonation (Fig. 4F).

### Comparison of ASFV 2′-O-MTase and VACV 2′-O-MTase

Subsequently, the VACV 2′-O-MTase VP39, an enzyme from an NCLDV member, was compared with the pEP424R of ASFV. Their sequence similarity is very low, and their amino acid identity is only 11.4% (Fig. S5A), and the structure of pEP424R is much more compact than that of VP39 (Fig. S5B and C).

**Fig 5:**
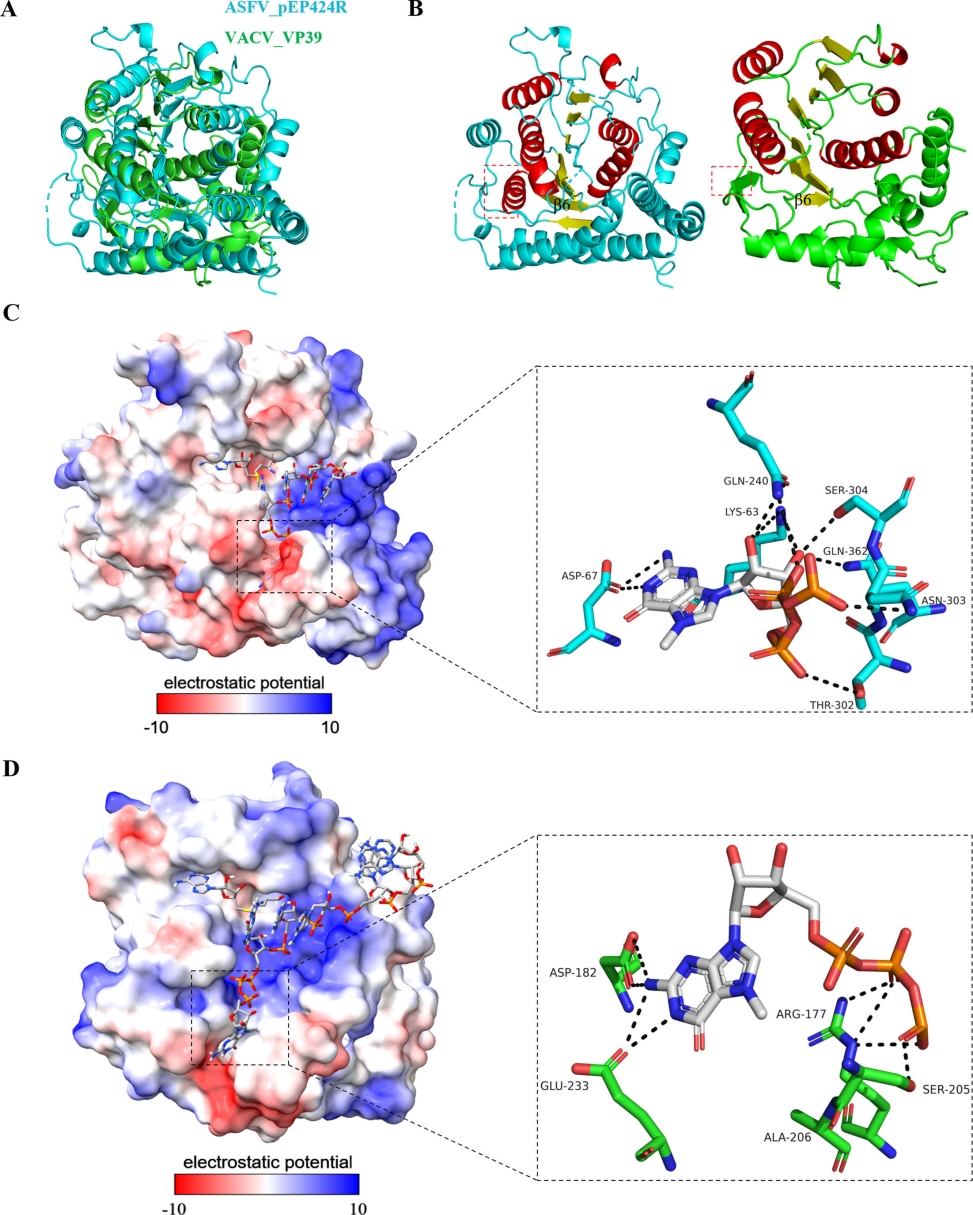
Structural comparison of ASFV 2′-O-MTase and VACV 2′-O-MTase. (**A**) Superimposition of pEP424R (cyan) on VP39 (green). (**B**) pEP424R and VP39 are presented in cartoon form. The α-helices in the central region are colored in red, and the β-sheets are colored in yellow. The red dotted box represents the difference between the two. (**C**) A model of RNA recognition by the ASFV pEP424R or VACV VP39. (**D**) Surface of pEP424R and VP39 is colored according to the electrostatic surface potential (left). Interaction of MGT with pEP424R or VP39 (right). The amino acid residues and small molecules shown are presented as usual.

The 3D structures of pEP424R and VP39 are also quite different (Fig. 5A). We found that VP39 did not have a complete Romance fold and that the alpha helix attached to β6 was replaced by a beta fold (Fig. 5B). By comparing the potential difference in the substrate binding complex between SAM and VP39, we found that the adenine position in the pEP424R binding pocket of Sam was mainly neutral, while the amino acid in VP39 was positively charged. Methionine is located at a position where the binding pocket of pEP424R is dominated by negatively charged amino acids, whereas the binding pocket of VP39 contains two positively and negatively charged amino acids (Fig. 5C). In addition, the MGT molecule of the methyl receptor is located in a canyon formed by polar amino acids in pEP424R and forms strong intermolecular interactions. MGT, on the other hand, is located on the surface of VP39, but it also interacts mostly with polar amino acids (Fig. 5D).

The similarities and differences between the above VP39 and pEP424R may be closely related to their functions. In VACV, VP39 not only functions as a 2′-O-MTase but also acts as a cofactor to facilitate the activity of the VACV poly(A) polymerase (38, 39). In contrast, the biological function of pEP424R remains unknown and requires further investigation.

### ASFV pEP424R is an early protein located mainly in the cytoplasm

**Fig 6:**
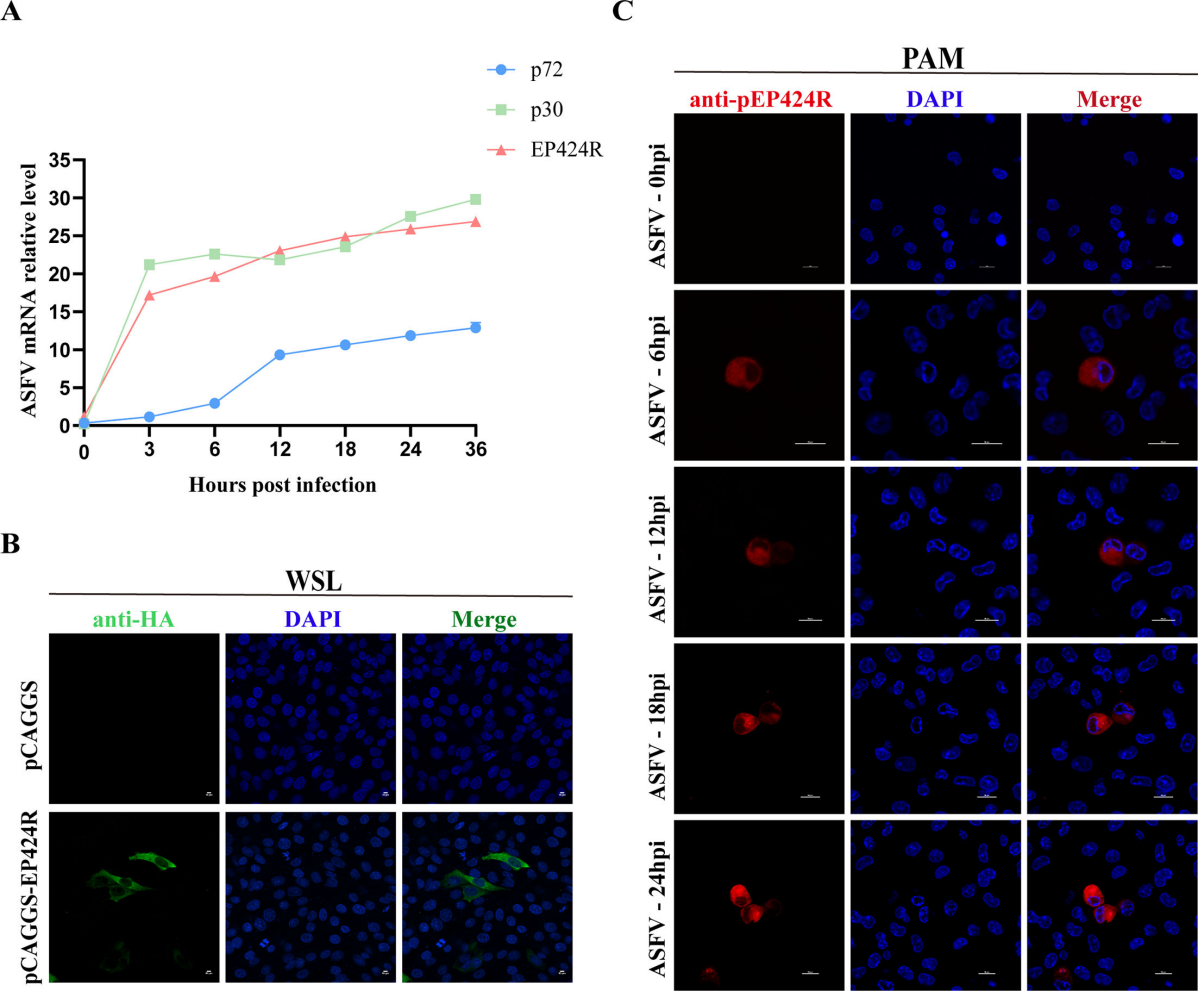
ASFV pEP424R is an early protein located mainly in the cytoplasm. (**A**) EP424R gene transcriptional dynamics. PAMs were infected with ASFV at an MOI of 1, and viral gene transcription was measured at 3, 6, 12, 18, 24, and 36 h post-infection by RT-qPCR. The expression levels of p30 and p72 were also measured as controls. (**B**) Intracellular localization of the ectopic expression of pEP424R. Wild boar lung (WSL) cells were transfected with pHA-EP424R for 24 h. The cells were fixed and probed with a mouse anti-HA monoclonal antibody and the nuclear marker 4,6-diamidino-2-phenylindole (DAPI) and then examined by laser confocal microscopy. Scale bars, 50 µm. (**C**) Subcellular localization of pEP424R in ASFV-infected cells. PAMs were infected with ASFV-WT at an MOI of 1 for 0, 6, 12, 18, and 24 h. The cells were fixed and incubated with homemade mouse anti-pEP424R polyclonal antibodies. The subcellular localization of pEP424R was visualized by laser confocal microscopy. Scale bars, 10 µm.

To investigate the transcription kinetics of *EP424R,* porcine alveolar macrophages (PAMs) infected with ASFV at a multiplicity of infection (MOI) of 1 were collected at 3, 6, 12, 18, 24, and 36 h post-infection (hpi). Reverse transcription-quantitative polymerase chain reaction (RT-qPCR) was used to compare *EP424R* mRNA levels, alongside early (*CP204L*, p30) and late (*B646L*, p72) ASFV genes (40). The results revealed that p30 and pEP424R had similar transcriptional kinetics and protein expression profiles, with both expressed at the early infection stage (6 hpi) (Fig. 6A).

To determine the subcellular localization of pEP424R in cells, WSL cells were transfected with pHA-EP424R expressing HA-tagged pEP424R in the pCAGGS vector and analyzed by laser confocal microscopy. The results revealed that this protein was evenly distributed in both the nucleus and the cytoplasm of the cells (Fig. 6B). To investigate the subcellular localization of pEP424R in ASFV-infected cells, PAMs were infected with ASFV at an MOI of 1 for 0, 6, 12, 18, and 24 h. The subcellular localization of pEP424R was visualized by an immunofluorescence assay using homemade mouse anti-pEP424R polyclonal antibodies (PAbs). The results indicated that pEP424R was minimally expressed in the nucleus from the early stages, with predominant localization in the cytoplasm. Notably, cytoplasmic pEP424R expression increased in a time-dependent manner, whereas no significant accumulation was detected in the nucleus (Fig. 6C). Furthermore, the subcellular localization of ASFV p72 has previously been documented within the viral factory (41). Immunofluorescence staining with homemade PAbs (anti-pEP424R in mouse and anti-p72 in rabbit) revealed clear co-localization of pEP424R with p72, indicating that pEP424R localizes to the viral factory (Fig. S6A).

### ASFV pEP424R is crucial for ASFV replication

**Fig 7:**
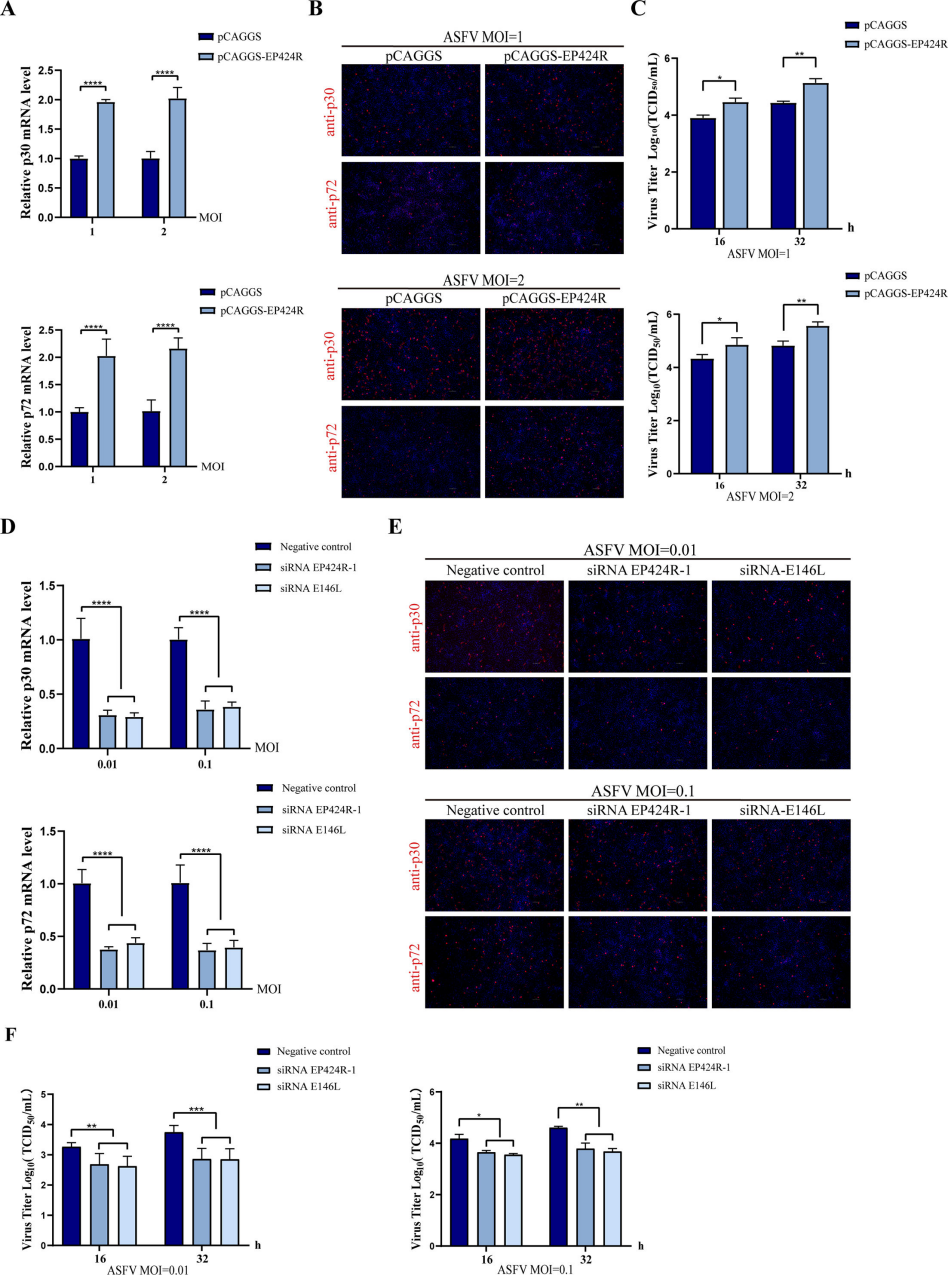
ASFV pEP424R is crucial for ASFV replication. (**A–C**) WSL cells were transfected with an empty vector plasmid or the pEP424R plasmid. At 24 h post-transfection, cells were infected with ASFV (MOI = 1 or 2) and collected at 24 hpi for RT-qPCR and IFA. At 24 h post-transfection, cells were infected with ASFV (MOI = 1 or 2) and collected at 16 and 32 hpi for TCID_50_ detection. Scale bar, 100 µm. (**D**) We transfected WSL cells with negative control (NC) small interfering RNA (siRNA), siRNA-EP424R-1, or siRNA-E146L and subsequently infected them with ASFV at MOIs of 0.01 or 0.1. The mRNA expression of p30 and p72 was measured by RT-qPCR at 24 hpi. (**E**) IFA of p30 and p72 protein in siRNA-treated WSL cells infected with ASFV (MOI = 0.01 or 0.1) at 24 hpi. Scale bar, 100 µm. (**F**) A reduction in viral yield was detected in ASFV-infected WSL cells (MOI = 0.01 or 0.1) transfected with siRNAs compared with controls at 16 and 32 hpi. Data are presented as the mean ± SD of the results from three independent experiments; ns (not significant), * (*P* < 0.05), ** (*P* < 0.01), and *** (*P* < 0.001).

To evaluate the role of pEP424R in the life cycle of ASFV, an overexpression assay was performed. First, WSL cells were transfected with pCAGGS or pCAGGS-EP424R for 24 h, respectively, and then infected with ASFV at an MOI of 1 or 2. Subsequent analyses using RT-qPCR, immunofluorescence assays (IFA), and 50% tissue culture infective dose (TCID_50_) experiments demonstrated that compared with the control treatment, pEP424R overexpression significantly elevated the mRNA and protein levels of p30 and p72 and increased ASFV titer (Fig. 7A through C). Among them, the overexpression of pEP424R at the mRNA and protein levels is shown in Fig. S7A and B. These results indicate that ASFV pEP424R significantly promotes the replication of ASFV.

To further investigate its role, WSL cells were transfected with three siRNAs targeting *EP424R*, and the knockdown efficiency in WSL cells was evaluated. Given its effective knockdown of EP424R in WSL cells according to RT-qPCR, siRNA EP424R-1 was selected for subsequent experiments (Fig. S7C). We selected the E146L gene knockdown as a positive control, as it has been reported to impair ASFV replication (42). To evaluate the knockdown efficiency of siRNA-E146L, we performed RT-qPCR to measure E146L mRNA levels (Fig. S7C). Furthermore, the mRNA expression of p30 and p72 was detected to assess ASFV replication (Fig. 7D). These findings are consistent with the IFA results, which showed that ASFV infection led to reduced expression of p30 and p72 in WSL cells following the knockdown of pEP424R or pE146L, respectively (Fig. 7E; Fig. S7D). Moreover, the results of the virus titer determination experiment were similar (Fig. 7F). These results indicate that pEP424R is crucial for ASFV replication. Knockdown of this protein impaired viral replication, whereas overexpression of this protein promoted viral growth.

**Fig 8:**
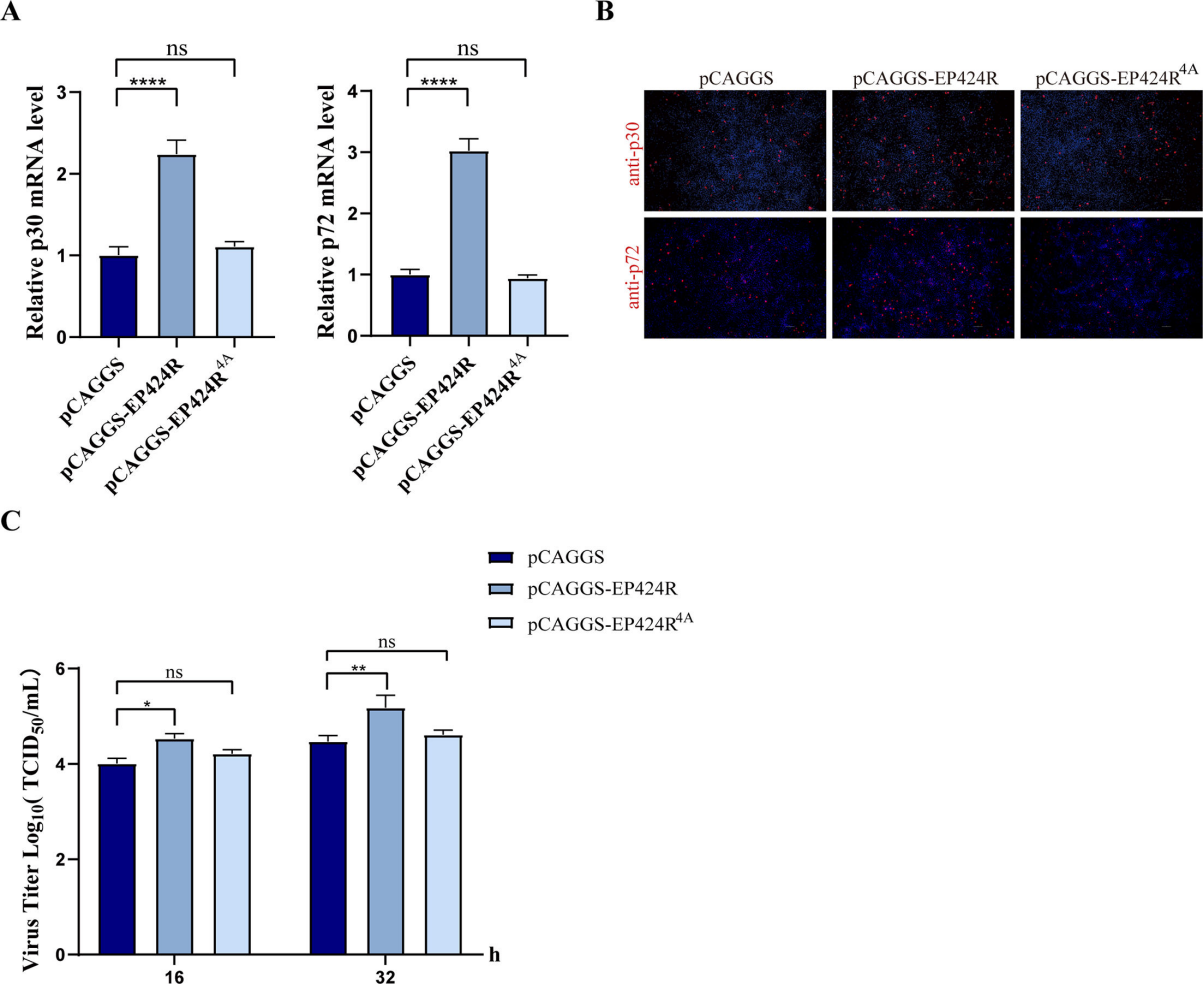
The promoting effect of ASFV pEP424R on viral proliferation involves its MTase activity. (**A–C**) WSL cells were transfected with empty vector plasmid, pEP424R plasmid, or pEP424R^4A^ plasmid. At 24 h post-transfection, cells were infected with ASFV (MOI = 1) and collected at 24 hpi for RT-qPCR and IFA. At 24 h post-transfection, cells were infected with ASFV (MOI = 1) and collected at 16 and 32 hpi for TCID_50_ detection. Scale bar, 100 µm. Data are presented as the mean ± SD of the results from three independent experiments; ns (not significant), * (*P* < 0.05), ** (*P* < 0.01), and *** (*P* < 0.001).

In addition, we constructed a recombinant eukaryotic expression plasmid (pCAGGS-EP424R^4A^) in which all the amino acids of the KDKE motif, which is the key enzymatic active site of pEP424R, were mutated to alanine. Consistent with the results of the aforementioned overexpression experiments, the results revealed that overexpression of pEP424R corresponded to an increase in ASFV p30 and p72 mRNA expression, whereas pCAGGS-EP424R^4A^ had no significant effect (Fig. 8A). Indirect IFA and virus titer determination also yielded consistent results (Fig. 8B and C). These data indicate that the overexpression of pEP424R promotes ASFV proliferation and that its function is to a certain extent related to its methyltransferase activity.

## DISCUSSION

ASFV causes a highly contagious and lethal disease in swine, for which there are currently no approved vaccines or antiviral drugs (1, 2). The capping of viral mRNA plays a pivotal role in gene expression, immune evasion, and viral replication (14–16). While the protein pNP868R has been identified as being involved in the formation of the cap-0 structure (11), the enzyme that catalyzes the conversion of cap-0 to cap-1 in ASFV mRNA has yet to be determined. In this study, we provide structural, biochemical, and functional evidence that *EP424R* encodes a 2′-O-MTase responsible for cap-1 formation, thereby completing the ASFV-encoded mRNA capping machinery.

First, from the perspective of protein structure elucidation, we obtained high-resolution crystal structures of pEP424R itself and its complexes with small-molecule compounds (SAM, SFG, SAH). Crystallographic analysis revealed that pEP424R adopts a classical Rossmann-fold structure, consisting of a central seven-β-sheet core flanked by six α-helices. Radioactive labeling enzyme activity assays demonstrated that pEP424R can transfer the methyl group from SAM to the 2′-hydroxyl group of the first nucleotide in the m⁷GpppA-RNA substrate. Further structural alignment and mutagenesis studies revealed that the residues critical for pEP424R activity, specifically the KDKE motif, are highly conserved among numerous viral 2′-O-MTases. Mutation of any single catalytic residue significantly impaired the enzymatic activity, a finding that underscores the critical role of these residues in the enzyme’s function. However, the most remarkable result was that the recombinant protein with mutations occurring simultaneously at all four sites exhibited almost complete loss of activity. This finding highlights the severe dependence of the enzyme’s function on these residues. These findings not only reveal the catalytic mechanism of pEP424R but also stress the severity of the enzyme’s dependence on the KDKE motif.

Notably, structural alignment revealed that the overall fold change of pEP424R has significant spatial consistency with that of the human 2′-O-MTase CMTR1 and SARS-CoV-2 nsp16, suggesting that its catalytic mechanism is evolutionarily convergent with that of homologous enzymes from eukaryotes and other viruses. This mechanistic conservation suggests that ASFV, as a large DNA virus that executes all post-transcriptional processing events within the cytoplasm, has independently evolved a complete and stable mRNA modification apparatus. This allows the virus to perform capping processes without relying on host enzymes. In contrast to the bifunctional enzymatic activity characteristics of VP39 in the related virus VACV (38, 39), pEP424R is significantly different in terms of molecular size, structural topology, and substrate charge distribution, which suggests that it has specific functions. These unique features of pEP424R have important implications for understanding viral evolution and approaches to antiviral development.

Our research further validated the function of pEP424R from the perspective of the viral life cycle. Transcriptional kinetic analysis revealed that pEP424R is significantly transcribed at the early stage of infection (6 hpi), with an expression pattern consistent with that of the known early gene p30. Combined with the results of subcellular localization, these findings suggest that pEP424R functions immediately after the initiation of viral transcription, promoting the stability and translation of early mRNA. Following transfection with pCAGGS-EP424R, we observed significant increases in the viral titer, gene transcription, and protein expression of ASFV. In siRNA experiments targeting ASFV *EP424R*, both RT-qPCR and IFA revealed that the expression of the ASFV p30 gene was downregulated. Additionally, viral titer experiments indicated that the knockdown of pEP424R impaired ASFV proliferation. These findings suggest that pEP424R plays a crucial role in regulating viral transcription and replication. Moreover, the results of the overexpression experiment on the pEP424R mutant plasmid (pCAGGS-EP424R^4A^) further confirm that the promotional effect of pEP424R overexpression on ASFV proliferation is, to a certain extent, related to its methyltransferase activity. Notably, these findings are consistent with the conclusions of studies on porcine epidemic diarrhea virus (PEDV) (43), which has significant implications for our understanding of ASFV gene function and its potential applications in virology.

Previous studies have confirmed that, for Middle East respiratory syndrome coronavirus and SARS-CoV, the NSP16-mutant virus (D130A) can induce a robust protective immune response *in vivo* following homologous virus challenge (44–46). With respect to PEDV, compared with the parental strain, the NSP16-mutant virus (PEDV-KDKE^4A^) has a significantly lower replicative capacity. Moreover, it can induce increased production of type I and type III interferons in infected cells. Challenge experiments have a survival rate of 100%, with an 80% protection rate against challenges with virulent strains (47). These findings indicate that nsp16 is a potential candidate vaccine target for coronaviruses and suggest that pEP424R may be a potential vaccine target for ASFV.

More importantly, this study highlights a specific molecular target for the development of anti-ASFV drugs. In recent years, research on the design of inhibitors targeting the active pocket of 2′-O-MTase has become a hot topic in the field. Virtual screening studies based on SARS-CoV-2 nsp16 have shown that small-molecule compounds such as framycetin, paromomycin, amikacin, hesperidin, and rimegepant are promising candidates for the treatment of SARS-CoV-2 infection (48, 49). Similarly, inhibitors developed based on the basis of MPXV VP39 have been confirmed to be effective antiviral drug targets. Both classical plaque assays and cell-based cytopathic effect assays have demonstrated that VP39 inhibitors can significantly suppress the proliferation of MPXV (50, 51). The pEP424R-SFG complex structure resolved in this study provides a valuable template for structure-based inhibitor screening. Chemical modification based on the SFG scaffold is expected to increase its affinity and selectivity for the active pocket of pEP424R. Therefore, the design of inhibitors targeting ASFV 2′-O-MTase is also an important research direction worthy of in-depth exploration.

**Fig 9:**
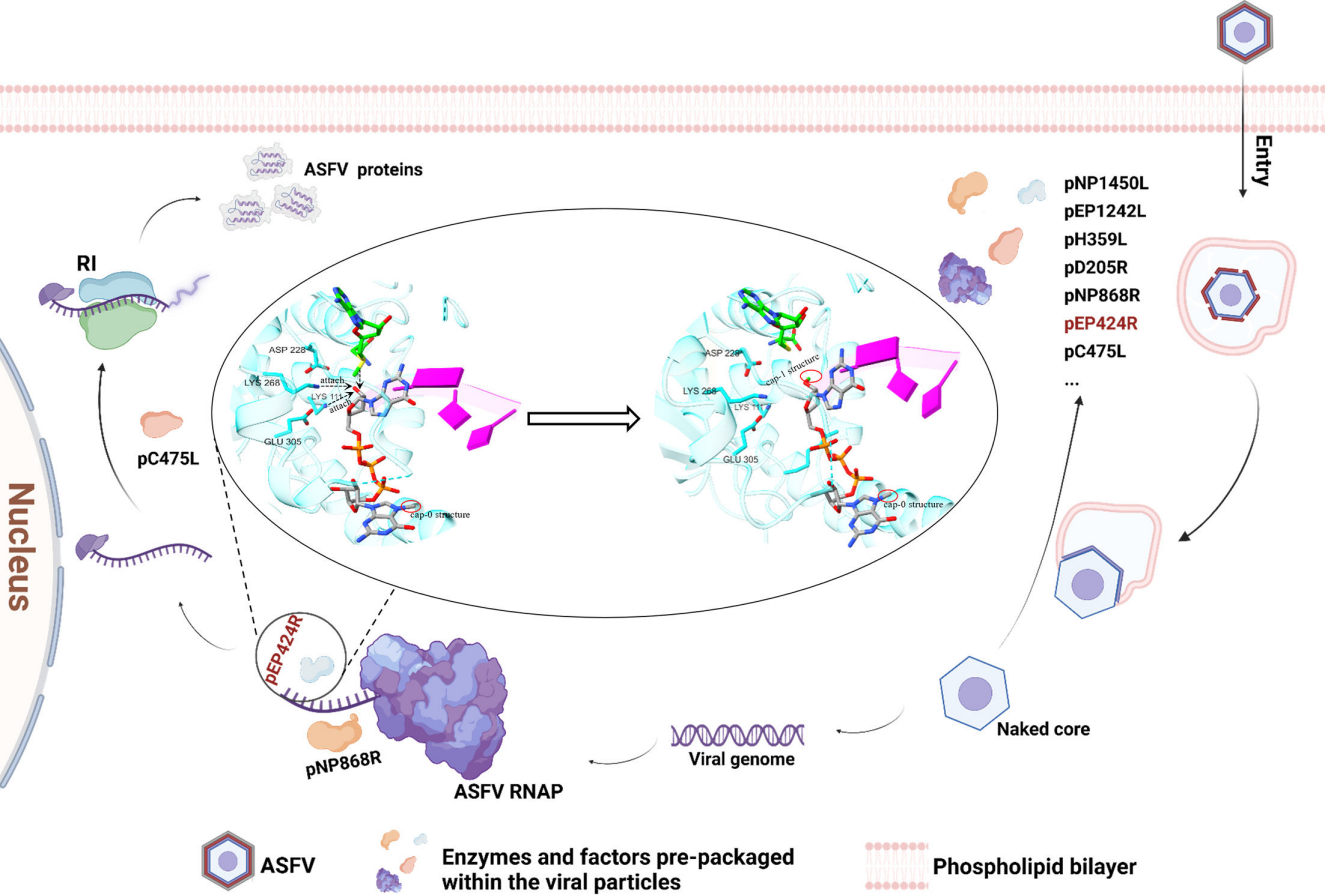
A model illustrating the role of pEP424R in the ASFV replication cycle is shown. During the ASFV infection process, the pEP424R encapsulated in the viral particles acts as a 2′-O-MTase to add a cap-1 structure to the ASFV genome, thereby ensuring the smooth progression of translation.

On the basis of this study, we propose a hypothetical model to illustrate the role of pEP424R in the life cycle of ASFV (Fig. 9). During ASFV infection, pEP424R, which is prepackaged in viral particles and functions as a 2′-O-MTase, can mediate the formation of cap-1 structure on the ASFV genome, thereby ensuring the smooth progression of the translation process. Furthermore, the results of virological studies indicate that pEP424R is crucial for ASFV replication and that its function is, to a certain extent, associated with its methyltransferase activity. In summary, this study not only represents the first identification of pEP424R as an mRNA cap-1 enzyme encoded by ASFV itself but also elaborates on its structure-function mechanism in depth and verifies its significance in the viral life cycle through a series of biochemical and virological experiments. pEP424R is not only a key node for understanding the mechanism that regulates ASFV gene expression but also an important molecular target for the future development of antiviral small-molecule drugs and attenuated vaccines. This discovery addresses a significant gap in the research on ASFV post-transcriptional processing and provides a new avenue for antiviral interventions aimed at the viral capping mechanism.

## MATERIALS AND METHODS

### Cells and viruses

Primary PAMs were harvested from the lungs of 4-week-old piglets as previously described (52). PAMs or WSL cells were grown in RPMI 1640 medium supplemented with 10% FBS. All the cell cultures were maintained at 37°C in a humidified incubator with 5% CO_2_.

The ASFV-HB01 strain was isolated and stored in the ABSL-3 laboratory of Huazhong Agricultural University.

### Plasmid construction

The ASFV *EP424R* gene was amplified by PCR with virus-derived cDNA fragments as the template using oligonucleotide primers. The amplified DNA fragment was purified, cloned, and inserted into the pET42b vector by homologous recombination. Furthermore, four single-point mutants (pEP424R-K111A, pEP424R-D228A, pEP424R-K268A, and pEP424R-E305A) and four point mutants (pEP424R-K111A/pEP424R-D228A/pEP424R-K268A/pEP424R-E305A) were introduced by designed primers with point mutations and PCR. The mutant pEP424R sequence was inserted into pET42b using the mentioned abovementioned method. The pET42b vector was digested with Nde I and Xho I (NEB) with eight histidine residues in the C-terminus. For eukaryotic expression, the *EP424R* fragment was cloned and inserted into a linearized pCAGGS vector (EcoRI and XhoI restriction sites; NEB) through homologous recombination. The pCAGGS vector was digested with EcoRI and XhoI (NEB) with an HA-tag at the N-terminus. The recombinant plasmid was transformed into an *Escherichia coli* DH5α cloning strain, which was subsequently plated onto lysogeny broth (LB) kanamycin plates. Single colonies were selected and sent to sequencing companies for sequencing. All primer sequences are listed in Table S1.

### Protein expression and purification

The correctly sequenced recombinant plasmid was transformed into *Escherichia coli* BL21 (DE3) strains, which were subsequently grown at 37°C in LB medium supplemented with 100 ug/mL kanamycin until they reached an optical density at 600 nm (OD600) of 0.6–0.8. Afterward, 1 mM isopropyl-β-D-thiogalactopyranoside was added to induce recombinant protein production for 20 h at 18°C. After induction, the strains were harvested by centrifugation at 4°C for 10 min at 8,500 rpm. The strain pellet was resuspended in lysis buffer (pH 7.4; 20 mM Tris-HCl; 500 mM NaCl; and 5 mM imidazole). The strains were lysed using an AH-1500 homogenizer (ATS Engineering Inc., Shanghai, China), and the lysate was centrifuged at 4°C for 1 h at 8,500 rpm. Afterward, the supernatant was filtered and loaded onto a His Trap TM HP column (GE Healthcare, Pittsburgh, USA). The target protein was pre-equilibrated with lysis buffer and eluted in gradient mixtures of binding buffer (pH 7.4; 20 mM Tris, 500 mM NaCl, and 500 mM imidazole), followed by elution with 30% to 100% elution buffer. The elution fraction was concentrated and further purified using a HiLoad 16/600 Superdex 200 pg column (GE Healthcare, Pittsburgh, USA) (120 mL) with 20 mM Tris-HCl and 200 mM NaCl (pH 7.4). All purification procedures were performed at 4°C to avoid protein degradation. The purified pEP424R was analyzed by SDS-PAGE. After analysis, the purest pEP424R was concentrated to 15 mg/mL, as determined by the absorbance at 280 nm. Finally, they were stored at −80°C for subsequent analysis.

### Protein crystallization and data collection

ASFV pEP424R was screened for crystallization by the sitting drop vapor diffusion method at 20°C. The crystallization conditions were optimized on the basis of the initial crystallization conditions. The optimal pEP424R crystal was obtained via vapor diffusion in sitting drops consisting of 1 µL of reservoir solution (0.2 M diammonium hydrogen citrate and 16% [wt/vol] PEG 33500) (Hampton Research, California, USA) and 1 µL of protein solution (15 mg/mL in 20 mM Tris-HCl and 200 mM NaCl; pH 7.4). Crystals of the pEP424R-SAM complex were grown in drops containing 1 µL of protein solution (15 mg/mL protein + 600 µm SAM + 8 mm MgCl_2_ incubated for 1 h at 4°C) and 1 µL of reservoir solution (0.2 M diammonium hydrogen citrate and 20% [wt/vol] PEG 33500). Crystals of the pEP424R-SFG complex were grown in drops containing 1 µL of protein solution (15 mg/mL protein + 600 µm SFG + 8 mm MgCl_2_ incubated for 1 h at 4°C) and 1 µL of reservoir solution (0.2 M diammonium hydrogen citrate and 15% [wt/vol] PEG 33500). Crystals of the pEP424R-SAH complex were grown in drops containing 1 µL of protein solution (11 mg/mL protein + 800 µm SAH + 8 mm MgCl_2_ incubated for 1 h at 4°C) and 1 µL of reservoir solution (0.2 M diammonium hydrogen citrate and 19% [wt/vol] PEG 33500).

The crystals were soaked in the reservoir solution supplemented with 25% (vol/vol) ethylene glycol for a few seconds and then flash-frozen in liquid nitrogen. X-ray diffraction data were collected at the BL10U2, BL02U1, and BL19U1 beamlines at the Shanghai Synchrotron Radiation Facility (SSRF) at cryogenic temperatures. The data collection statistics are shown in Table 1.

### Crystal structure determination and refinement

All the data were processed by HKL2000 (53). The initial phases were calculated using the program Phaser (54), with the structure predicted by AlphaFold2 (34) used as the search model. The structure was refined with the program Phenix (55) and manually corrected in Coot (56). Structural diagrams were drawn using PYMOL (https://pymol.org/2/) and Chimera (57). The refinement statistics and model parameters are shown in Table 1.

### Electrophoretic mobility shift assay

EMSA was performed as described previously (58). Briefly, pEP424R at various concentrations was incubated with 1 µm Cy5-labeled nucleic acid (ssRNA, dsRNA, ssDNA, and dsDNA) in interaction buffer (200 mM NaCl, 10 mM Tris [pH 8.0], and 10% glycerol) for 45 min in the dark at room temperature. The samples were then electrophoresed on a 6.5% native gel at 180 V for 37 min, and the results were observed using an FLA-2000 fluorescence image analyzer.

### Biolayer interferometry assay

The binding of pEP424R to nucleic acids (ssRNA, dsRNA, ssDNA, and dsDNA) was analyzed as described previously (59). The difference is that we performed biotin labeling on the nucleic acids. One micromolar biotin-labeled nucleic acid was immobilized on SA biosensors (Octet, Sartorius) using a black 96-well plate (Greiner Bio-One) for BLI detection. All the experiments were performed at 25°C with agitation (e.g., 1,000 rpm), and accurate *Kd* values were calculated using Data Analysis Software 11.0. The nucleic acid fragments used for ESMA and BLI are listed in Table S2.

### *In vitro* MTase assay

The MTase assay methods used in this study were as previously described (60). Protein expression and purification were performed as described above. Synthetic RNA substrates in vitro were capped separately. ASFV 2′-O-MTase activity was assayed by incubating ASFV MTase with m7GpppA-RNA-capped RNA substrates. Moreover, the nsp16/nsp10 of FIPV was used as a positive control, while a sample without added protein was used as the negative control. The amount of radioactivity transferred onto the RNA was measured using a liquid scintillation counter (Tri-Carb 2910TR). Each sample was run three times to ensure accuracy.

### Viral infection and RT-qPCR

To investigate the transcriptional level of the *EP424R* gene in PAMs during ASFV-HB01 infection, total RNA was extracted from infected cells (MOI = 1) at 3, 6, 12, 18, 24, and 36 hpi. The expression levels were then analyzed via RT-qPCR as previously described (61). ASFV P30 and P72 were used as controls in the experiments. The qPCR primers used are listed in Table S1.

### Mouse immunization

Six-week-old female BALB/c mice were immunized intramuscularly on days 0 and 21 with 50 μL of a 1:1 dilution of purified pEP424R (50 µg/mouse) adjuvanted with QuickAntibody-Mouse5W (BIODRAGON, Beijing, China). Two weeks after the final immunization, serum was collected for subsequent assays.

### Transfection

WSL cells were grown to 80% confluence in RPMI 1640 medium supplemented with 10% FBS and 1% penicillin‒streptomycin. Cells were then transfected with JETPRIME (POLYPLUS) reagent according to the manufacturer’s protocol. The small interfering RNAs used in this study are listed in Table S3.

### Immunostaining and confocal microscopy

Transfected cells were washed with PBS 24 h post-transfection, fixed with 4% paraformaldehyde at room temperature for 30 min, and permeabilized with 0.2% Triton X-100 for 10 min. After three washes with PBS, the cells were blocked with PBS containing 5% bovine serum albumin for 1 h at room temperature. The cells were then incubated with primary antibodies at 37°C, followed by incubation with Alexa-labeled secondary antibodies (Invitrogen, A-11005; 1:1,000). Nuclei were stained with DAPI (Sigma, D9542) for 5 min at room temperature. Images were captured using a confocal microscope (Zeiss LSM 800) with a 63× oil immersion objective and analyzed using ZEN microscopy software (Zeiss).

For infection experiments, cells were washed with PBS for the indicated times post-infection, and the same immunostaining protocol was applied to the cells. The primary antibodies used included ASFV pEP424R (a mouse anti-ASFV pEP424R polyclonal antibody, prepared in-house), p72 (a mouse anti-ASFV p72 polyclonal antibody, prepared in-house), and p30 (a mouse anti-ASFV p30 polyclonal antibody, prepared in-house). The samples were imaged using a fluorescence microscope (Nikon Ti2-A) or a laser scanning confocal microscope (Zeiss LSM 800).

### Viral titers

PAMs or WSL cells were inoculated with ASFV in 24-well plates in triplicate wells. Infected cells were harvested together with culture supernatants at the indicated time points post-infection, and viral titers were determined by a TCID_50_ assay. Specifically, the collected supernatants were 10-fold serially diluted and added to 96-well plates pre-seeded with PAMs. Following incubation at 37°C with 5% CO₂ for 3–5 days, the cell plates were processed according to the immunofluorescence assay protocol, using a p30-specific antibody to label infected cells. Viral titers were subsequently observed by fluorescence microscopy and calculated.

### Statistical analysis

Unpaired two-tailed t tests were used to determine statistical significance, with analyses performed using GraphPad Prism version 8.0 (GraphPad Software, San Diego, California, USA). The data are presented as the mean ± SD from three independent experiments. After confirming a normal distribution and homogeneity of variance, *P* ≥ 0.05 was to indicate statistical non-significance (ns); *P* < 0.05 was considered to indicate statistical significance (*P < 0.05, **P < 0.01, ***P < 0.001, ****P < 0.0001).

## Data Availability

Structural data were deposited in the RCSB Protein Data Bank (PDB) under accession codes 9W69, 9W7G, 9W7F, and 9IQ4. The data that support the findings of this study are openly available in this article and are available from the corresponding author upon request.

## SUPPLEMENTAL MATERIAL

Supplemental figures and tables mbio.02786-25-s0001.docx Fig. S1 to S7; Tables S1 to S3.

ASM does not own the copyrights to Supplemental Material that may be linked to, or accessed through, an article. The authors have granted ASM a non-exclusive, world-wide license to publish the Supplemental Material files. Please contact the corresponding author directly for reuse.

## References

[1] Dixon LK, Sun H, Roberts H. 2019. African swine fever. Antiviral Res 165:34–41. doi:10.1016/j.antiviral.2019.02.018 30836106

[2] Galindo I, Alonso C. 2017. African swine fever virus: a review. Viruses 9:103. doi:10.3390/v9050103 28489063 PMC5454416

[3] Eustace Montgomery R. 1921. On a form of swine fever occurring in British East Africa (Kenya Colony). J Comp Pathol Ther 34:159–191. doi:10.1016/S0368-1742(21)80031-4

[4] Sánchez-Cordón PJ, Montoya M, Reis AL, Dixon LK. 2018. African swine fever: a re-emerging viral disease threatening the global pig industry. Vet J 233:41–48. doi:10.1016/j.tvjl.2017.12.025 29486878 PMC5844645

[5] Karger A, Pérez-Núñez D, Urquiza J, Hinojar P, Alonso C, Freitas FB, Revilla Y, Le Potier M-F, Montoya M. 2019. An update on african swine fever virology. Viruses 11:864. doi:10.3390/v11090864 31533244 PMC6784044

[6] Liang R, Wang G, Zhang D, Ye G, Li M, Shi Y, Shi J, Chen H, Peng G. 2021. Structural comparisons of host and African swine fever virus dUTPases reveal new clues for inhibitor development. J Biol Chem 296:100015. doi:10.1074/jbc.RA120.014005 33139328 PMC7948977

[7] Urbano AC, Ferreira F. 2022. African swine fever control and prevention: an update on vaccine development. Emerg Microbes Infect 11:2021–2033. doi:10.1080/22221751.2022.2108342 35912875 PMC9423837

[8] Wu K, Liu J, Wang L, Fan S, Li Z, Li Y, Yi L, Ding H, Zhao M, Chen J. 2020. Current state of global african swine fever vaccine development under the prevalence and transmission of ASF in China. Vaccines (Basel) 8:531. doi:10.3390/vaccines8030531 32942741 PMC7564663

[9] Zhang H, Zhao S, Zhang H, Qin Z, Shan H, Cai X. 2023. Vaccines for African swine fever: an update. Front Microbiol 14:1139494. doi:10.3389/fmicb.2023.1139494 37180260 PMC10173882

[10] Ramanathan A, Robb GB, Chan S-H. 2016. mRNA capping: biological functions and applications. Nucleic Acids Res 44:7511–7526. doi:10.1093/nar/gkw551 27317694 PMC5027499

[11] Du X, Gao Z-Q, Geng Z, Dong Y-H, Zhang H. 2021. Structure and biochemical characteristic of the methyltransferase (MTase) domain of RNA capping enzyme from African swine fever virus. J Virol 95:e02029–20, doi:10.1128/JVI.02029-20 33268516 PMC8092831

[12] Nallagatla SR, Toroney R, Bevilacqua PC. 2008. A brilliant disguise for self RNA: 5’-end and internal modifications of primary transcripts suppress elements of innate immunity. RNA Biol 5:140–144. doi:10.4161/rna.5.3.6839 18769134 PMC2809118

[13] Rehwinkel J, Tan CP, Goubau D, Schulz O, Pichlmair A, Bier K, Robb N, Vreede F, Barclay W, Fodor E, Reis e Sousa C. 2010. RIG-I detects viral genomic RNA during negative-strand RNA virus infection. Cell 140:397–408. doi:10.1016/j.cell.2010.01.020 20144762

[14] Benarroch D, Smith P, Shuman S. 2008. Characterization of a trifunctional mimivirus mRNA capping enzyme and crystal structure of the RNA triphosphatase domain. Struct Lond Engl 16:501–512. doi:10.1016/j.str.2008.01.009 18400173

[15] Decroly E, Ferron F, Lescar J, Canard B. 2011. Conventional and unconventional mechanisms for capping viral mRNA. Nat Rev Microbiol 10:51–65. doi:10.1038/nrmicro2675 22138959 PMC7097100

[16] Furuichi Y, Shatkin AJ. 2000. Viral and cellular mRNA capping: past and prospects. Adv Virus Res 55:135–184. doi:10.1016/s0065-3527(00)55003-9 11050942 PMC7131690

[17] Shuman S. 2015. RNA capping: progress and prospects. RNA 21:735–737. doi:10.1261/rna.049973.115 25780214 PMC4371356

[18] Ghosh A, Lima CD. 2010. Enzymology of RNA cap synthesis. Wiley Interdiscip Rev RNA 1:152–172. doi:10.1002/wrna.19 21956912 PMC3962952

[19] Russ A, Wittmann S, Tsukamoto Y, Herrmann A, Deutschmann J, Lagisquet J, Ensser A, Kato H, Gramberg T. 2022. Nsp16 shields SARS-CoV-2 from efficient MDA5 sensing and IFIT1-mediated restriction. EMBO Rep 23:e55648. doi:10.15252/embr.202255648 36285486 PMC9724656

[20] Schnierle BS, Gershon PD, Moss B. 1992. Cap-specific mRNA (nucleoside-O2’-)-methyltransferase and poly(A) polymerase stimulatory activities of vaccinia virus are mediated by a single protein. Proc Natl Acad Sci USA 89:2897–2901. doi:10.1073/pnas.89.7.2897 1313572 PMC48770

[21] Shuman S. 2002. What messenger RNA capping tells us about eukaryotic evolution. Nat Rev Mol Cell Biol 3:619–625. doi:10.1038/nrm880 12154373

[22] Kelly BJ, King LA, Possee RD. 2016. Introduction to baculovirus molecular biology. Methods Mol Biol Clifton NJ 1350:25–50. doi:10.1007/978-1-4939-3043-2_2 26820852

[23] Zhao D, Wang N, Feng X, Zhang Z, Xu K, Zheng T, Yang Y, Li X, Ou X, Zhao R, Rao Z, Bu Z, Chen Y, Wang X. 2024. Transcription regulation of African swine fever virus: dual role of M1249L. Nat Commun 15:10058. doi:10.1038/s41467-024-54461-1 39567541 PMC11579359

[24] Reis AL, Netherton C, Dixon LK. 2017. Unraveling the armor of a killer: evasion of host defenses by African swine fever virus. J Virol 91:e02338-16. doi:10.1128/JVI.02338-16 28031363 PMC5331812

[25] Cackett G, Sýkora M, Werner F. 2020. Transcriptome view of a killer: African swine fever virus. Biochem Soc Trans 48:1569–1581. doi:10.1042/BST20191108 32725217 PMC7458399

[26] Zhang Y, Zhang Z, Zhang F, Zhang J, Jiao J, Hou M, Qian N, Zhao D, Zheng X, Tan X. 2023. ASFV transcription reporter screening system identifies ailanthone as a broad antiviral compound. Virol Sin 38:459–469. doi:10.1016/j.virs.2023.03.004 36948461 PMC10311270

[27] Pilotto S, Sýkora M, Cackett G, Dulson C, Werner F. 2024. Structure of the recombinant RNA polymerase from African swine fever virus. Nat Commun 15:1606. doi:10.1038/s41467-024-45842-7 38383525 PMC10881513

[28] Zhu G, Xi F, Zeng W, Zhao Y, Cao W, Liu C, Yang F, Ru Y, Xiao S, Zhang S, et al.. 2025. Structural basis of RNA polymerase complexes in African swine fever virus. Nat Commun 16:501. doi:10.1038/s41467-024-55683-z 39779680 PMC11711665

[29] Rodríguez JM, Salas ML. 2013. African swine fever virus transcription. Virus Res 173:15–28. doi:10.1016/j.virusres.2012.09.014 23041356

[30] Corbett AH. 2018. Post-transcriptional regulation of gene expression and human disease. Curr Opin Cell Biol 52:96–104. doi:10.1016/j.ceb.2018.02.011 29518673 PMC5988930

[31] Alejo A, Matamoros T, Guerra M, Andrés G. 2018. A proteomic atlas of the African swine fever virus particle. J Virol 92:e01293-18. doi:10.1128/JVI.01293-18 30185597 PMC6232493

[32] Iyer LM, Balaji S, Koonin EV, Aravind L. 2006. Evolutionary genomics of nucleo-cytoplasmic large DNA viruses. Virus Res 117:156–184. doi:10.1016/j.virusres.2006.01.009 16494962

[33] Dixon LK, Chapman DAG, Netherton CL, Upton C. 2013. African swine fever virus replication and genomics. Virus Res 173:3–14. doi:10.1016/j.virusres.2012.10.020 23142553

[34] Jumper J, Evans R, Pritzel A, Green T, Figurnov M, Ronneberger O, Tunyasuvunakool K, Bates R, Žídek A, Potapenko A, et al.. 2021. Highly accurate protein structure prediction with AlphaFold. Nature 596:583–589. doi:10.1038/s41586-021-03819-2 34265844 PMC8371605

[35] Krissinel E, Henrick K. 2004. Secondary-structure matching (SSM), a new tool for fast protein structure alignment in three dimensions. Acta Crystallogr D Biol Crystallogr 60:2256–2268. doi:10.1107/S0907444904026460 15572779

[36] Bélanger F, Stepinski J, Darzynkiewicz E, Pelletier J. 2010. Characterization of hMTr1, a human Cap1 2’-O-ribose methyltransferase. J Biol Chem 285:33037–33044. doi:10.1074/jbc.M110.155283 20713356 PMC2963352

[37] Smietanski M, Werner M, Purta E, Kaminska KH, Stepinski J, Darzynkiewicz E, Nowotny M, Bujnicki JM. 2014. Structural analysis of human 2’-O-ribose methyltransferases involved in mRNA cap structure formation. Nat Commun 5:3004. doi:10.1038/ncomms4004 24402442 PMC3941023

[38] Mao X, Shuman S. 1994. Intrinsic RNA (guanine-7) methyltransferase activity of the vaccinia virus capping enzyme D1 subunit is stimulated by the D12 subunit. Identification of amino acid residues in the D1 protein required for subunit association and methyl group transfer. J Biol Chem 269:24472–24479. 7929111

[39] Schwer B, Hausmann S, Schneider S, Shuman S. 2006. Poxvirus mRNA cap methyltransferase. bypass of the requirement for the stimulatory subunit by mutations in the catalytic subunit and evidence for intersubunit allostery. J Biol Chem 281:18953–18960. doi:10.1074/jbc.M602867200 16707499

[40] Zheng Y, Li S, Li S-H, Yu S, Wang Q, Zhang K, Qu L, Sun Y, Bi Y, Tang F, Qiu H-J, Gao GF. 2022. Transcriptome profiling in swine macrophages infected with African swine fever virus at single-cell resolution. Proc Natl Acad Sci USA 119:e2201288119. doi:10.1073/pnas.2201288119 35507870 PMC9171760

[41] Castelló A, Quintas A, Sánchez EG, Sabina P, Nogal M, Carrasco L, Revilla Y. 2009. Regulation of host translational machinery by African swine fever virus. PLoS Pathog 5:e1000562. doi:10.1371/journal.ppat.1000562 19714237 PMC2727446

[42] Guo Y, Niu S, Wang X, Wang Z, Liang R, Tan Y, Fu Z, Su Z, Xu J, Chen H, Shi Y, Sun L, Peng G. 2025. ASFV pE146L-induced ER remodeling is essential for viral replication. J Virol 99:e0083425. doi:10.1128/jvi.00834-25 40767480 PMC12455986

[43] Shi P, Su Y, Li R, Liang Z, Dong S, Huang J. 2019. PEDV nsp16 negatively regulates innate immunity to promote viral proliferation. Virus Res 265:57–66. doi:10.1016/j.virusres.2019.03.005 30849413 PMC7114654

[44] Menachery VD, Gralinski LE, Mitchell HD, Dinnon KH III, Leist SR, Yount BL, Graham RL, McAnarney ET, Stratton KG, Cockrell AS, Debbink K, Sims AC, Waters KM, Baric RS. 2017. Middle east respiratory syndrome coronavirus nonstructural protein 16 is necessary for interferon resistance and viral pathogenesis. mSphere 2:e00346–17. doi:10.1128/mSphere.00346-17 29152578 PMC5687918

[45] Menachery V.D, Yount BL, Josset L, Gralinski LE, Scobey T, Agnihothram S, Katze MG, Baric RS. 2014. Attenuation and restoration of severe acute respiratory syndrome coronavirus mutant lacking 2’-o-methyltransferase activity. J Virol 88:4251–4264. doi:10.1128/JVI.03571-13 24478444 PMC3993736

[46] Menachery VD, Gralinski LE, Mitchell HD, Dinnon KH III, Leist SR, Yount BL, McAnarney ET, Graham RL, Waters KM, Baric RS. 2018. Combination attenuation offers strategy for live attenuated coronavirus vaccines. J Virol 92:e00710–18. doi:10.1128/JVI.00710-18 29976657 PMC6096805

[47] Hou Y, Ke H, Kim J, Yoo D, Su Y, Boley P, Chepngeno J, Vlasova AN, Saif LJ, Wang Q. 2019. Engineering a live attenuated porcine epidemic diarrhea virus vaccine candidate via inactivation of the viral 2’-O-methyltransferase and the endocytosis signal of the spike protein. J Virol 93:e00406-19. doi:10.1128/JVI.00406-19 31118255 PMC6639265

[48] Jiang Y, Liu L, Manning M, Bonahoom M, Lotvola A, Yang Z, Yang Z-Q. 2022. Structural analysis, virtual screening and molecular simulation to identify potential inhibitors targeting 2’-O-ribose methyltransferase of SARS-CoV-2 coronavirus. J Biomol Struct Dyn 40:1331–1346. doi:10.1080/07391102.2020.1828172 33016237 PMC7544923

[49] Kumar S, Singh H, Prajapat M, Sarma P, Bhattacharyya A, Kaur H, Kaur G, Shekhar N, Kaushal K, Kumari K, Bansal S, Mahendiratta S, Chauhan A, Singh A, Soloman Singh R, Sharma S, Thota P, Avti P, Prakash A, Kuhad A, Medhi B. 2023. Structural-based virtual screening of FDA-approved drugs repository for NSP16 inhibitors, essential for SARS-COV-2 invasion into host cells: elucidation from MM/PBSA calculation. Bioinform Biol Insights 17:11779322231171777. doi:10.1177/11779322231171777 37533429 PMC10392196

[50] Silhan J, Klima M, Otava T, Skvara P, Chalupska D, Chalupsky K, Kozic J, Nencka R, Boura E. 2023. Discovery and structural characterization of monkeypox virus methyltransferase VP39 inhibitors reveal similarities to SARS-CoV-2 nsp14 methyltransferase. Nat Commun 14:2259. doi:10.1038/s41467-023-38019-1 37080993 PMC10116469

[51] Zgarbová M, Otava T, Silhan J, Nencka R, Weber J, Boura E. 2023. Inhibitors of mpox VP39 2’-O methyltransferase efficiently inhibit the monkeypox virus. Antiviral Res 218:105714. doi:10.1016/j.antiviral.2023.105714 37689311

[52] Wardley RC, Wilkinson PJ. 1978. The growth of virulent African swine fever virus in pig monocytes and macrophages. Journal of General Virology 38:183–186. doi:10.1099/0022-1317-38-1-183 340610

[53] Otwinowski Z, Minor W. 1997. Processing of X-ray diffraction data collected in oscillation mode. Methods Enzymol 276:307–326. doi:10.1016/S0076-6879(97)76066-X 27754618

[54] McCoy AJ, Grosse-Kunstleve RW, Adams PD, Winn MD, Storoni LC, Read RJ. 2007. Phaser crystallographic software. J Appl Crystallogr 40:658–674. doi:10.1107/S0021889807021206 19461840 PMC2483472

[55] Adams PD, Afonine PV, Bunkóczi G, Chen VB, Davis IW, Echols N, Headd JJ, Hung L-W, Kapral GJ, Grosse-Kunstleve RW, McCoy AJ, Moriarty NW, Oeffner R, Read RJ, Richardson DC, Richardson JS, Terwilliger TC, Zwart PH. 2010. PHENIX: a comprehensive Python-based system for macromolecular structure solution. Acta Crystallogr D Biol Crystallogr 66:213–221. doi:10.1107/S0907444909052925 20124702 PMC2815670

[56] Emsley P, Lohkamp B, Scott WG, Cowtan K. 2010. Features and development of Coot. Acta Crystallogr D Biol Crystallogr 66:486–501. doi:10.1107/S0907444910007493 20383002 PMC2852313

[57] Pettersen EF, Goddard TD, Huang CC, Couch GS, Greenblatt DM, Meng EC, Ferrin TE. 2004. UCSF Chimera--a visualization system for exploratory research and analysis. J Comput Chem 25:1605–1612. doi:10.1002/jcc.20084 15264254

[58] Min B, Collins K. 2009. An RPA-related sequence-specific DNA-binding subunit of telomerase holoenzyme is required for elongation processivity and telomere maintenance. Mol Cell 36:609–619. doi:10.1016/j.molcel.2009.09.041 19941821 PMC2913470

[59] Zheng M, Feng B, Zhang Y, Liu X, Zhao N, Liu H, Xu Z, He X, Qu Z, Chen S, Jiang Z, Cheng X, Liu H, Zang Y, Zhao L, Zheng J, Zhang L, Li J, Zhou Y. 2024. Discovery and characterization of novel potent non-covalent small molecule inhibitors targeting papain-like protease from SARS-CoV-2. Acta Pharm Sin B 14:3286–3290. doi:10.1016/j.apsb.2024.04.011 39027261 PMC11252453

[60] Wang Y, Sun Y, Wu A, Xu S, Pan R, Zeng C, Jin X, Ge X, Shi Z, Ahola T, Chen Y, Guo D. 2015. Coronavirus nsp10/nsp16 methyltransferase can be targeted by nsp10-derived peptide in vitro and in vivo to reduce replication and pathogenesis. J Virol 89:8416–8427. doi:10.1128/JVI.00948-15 26041293 PMC4524257

[61] Liang R, Fu Y, Li G, Shen Z, Guo F, Shi J, Guo Y, Zhang D, Wang Z, Chen C, Shi Y, Peng G. 2024. EP152R-mediated endoplasmic reticulum stress contributes to African swine fever virus infection via the PERK-eIF2α pathway. FASEB J Off Publ Fed Am Soc Exp Biol 38:e70187. doi:10.1096/fj.202400931RR 39560029

