# Supplementary material for: The EP424R protein of African swine fever virus functions as a 2′-O-methyltransferase and plays an important role in viral replication
Source: mBio. 2026 Mar 6;17(4):e02786-25. doi: 10.1128/mbio.02786-25 (PMC13059714; doi:10.1128/mbio.02786-25)
Supplement: Supplemental figures and tables — Fig. S1 to S7; Tables S1 to S3. [file mbio.02786-25-s0001.docx]

**Supplementary Materials for**

**The EP424R protein of African swine fever virus functions as a 2'-O-methyltransferase and plays an important role in viral replication**

Zixuan Wang^1,2#^, Fenglin Guo^1,2#^, Xueying Wang^1,2^, Lin Cheng^1,2^, Yan Liu^1,2^, Yilin Guo^1,2^, Sai Niu^1,2^, Hakimeh Baghaei Daemi^1,2^, Guiqing Peng^1,2,3✉^

^1^State Key Laboratory of Agricultural Microbiology, College of Veterinary Medicine, Huazhong Agricultural University, Wuhan, China

^2^Key Laboratory of Preventive Veterinary Medicine in Hubei Province, The Cooperative Innovation Center for Sustainable Pig Production, Wuhan, China

^3^Key Laboratory of Prevention & Control for African Swine Fever and Other Major Pig Diseases, Ministry of Agriculture and Rural Affairs, Wuhan, China

^✉^To whom correspondence should be addressed: Guiqing Peng: State Key Laboratory of Agricultural Microbiology, College of Veterinary Medicine, Huazhong Agricultural University, No.1 Shizishan Street, Hongshan District, Wuhan, Hubei Province, 430070, P. R. China. Tel: +86-18071438015; Fax: 086-027-87280480; Email: [penggq@mail.hzau.edu.cn](mailto:penggq@mail.hzau.edu.cn)

^#^Zixuan Wang and Fenglin Guo contributed equally to this work and share first authorship. Author order was determined by drawing straws.

The authors declare no conflict of interest.

^✉^Corresponding author.

^#^Contributed equally.

**This PDF file includes:**

Figures S1 to S7

Tables S1 to S3


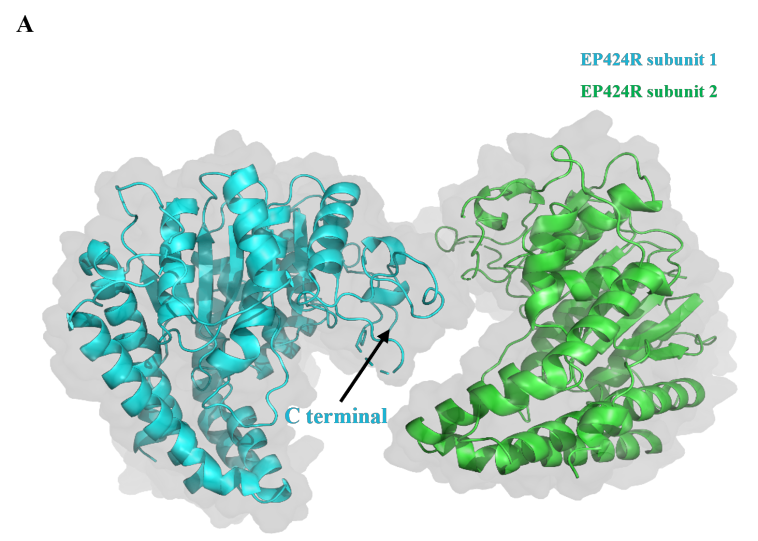


**Figure S1. Structure of ASFV pEP424R**

(A) Resolved dimer crystal structure of pEP424R. Subunits 1 and 2 are colored cyan and green, respectively.


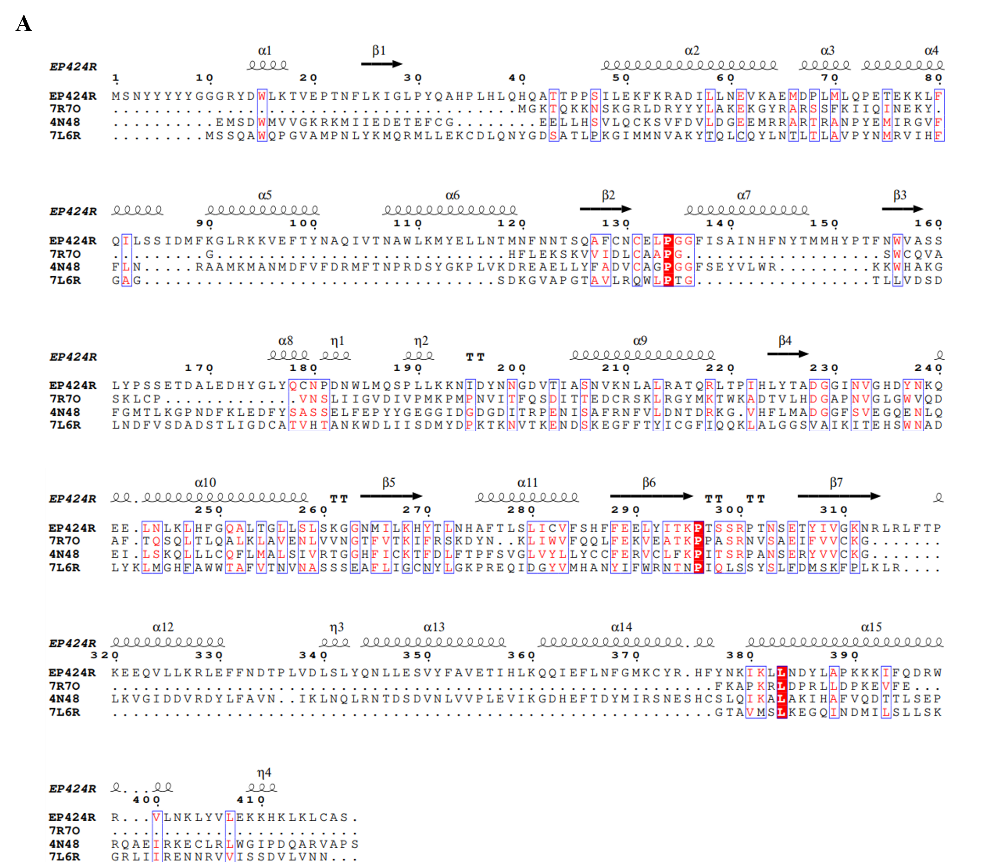


**Figure S2. Amino acid sequence ratio comparisons based on proteins structurally similar to *EP424R***

(A) The secondary structures of *EP424R* are presented at the top of the sequences. The identical conserved and low conserved residues are highlighted by a red background and red letters, respectively. Similar residues are in blue boxes.


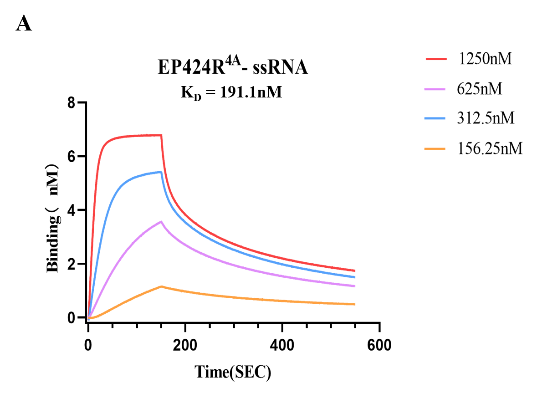


**Figure S3. Comparison of the active pocket before and after pEP424R exerted the enzymatic active function**

(A) BLI was performed to analyze the binding kinetics between pEP424R and nucleic acids. One micromolar biotin-labeled nucleic acid was immobilized on SA biosensors and incubated with twofold serially diluted pEP424R


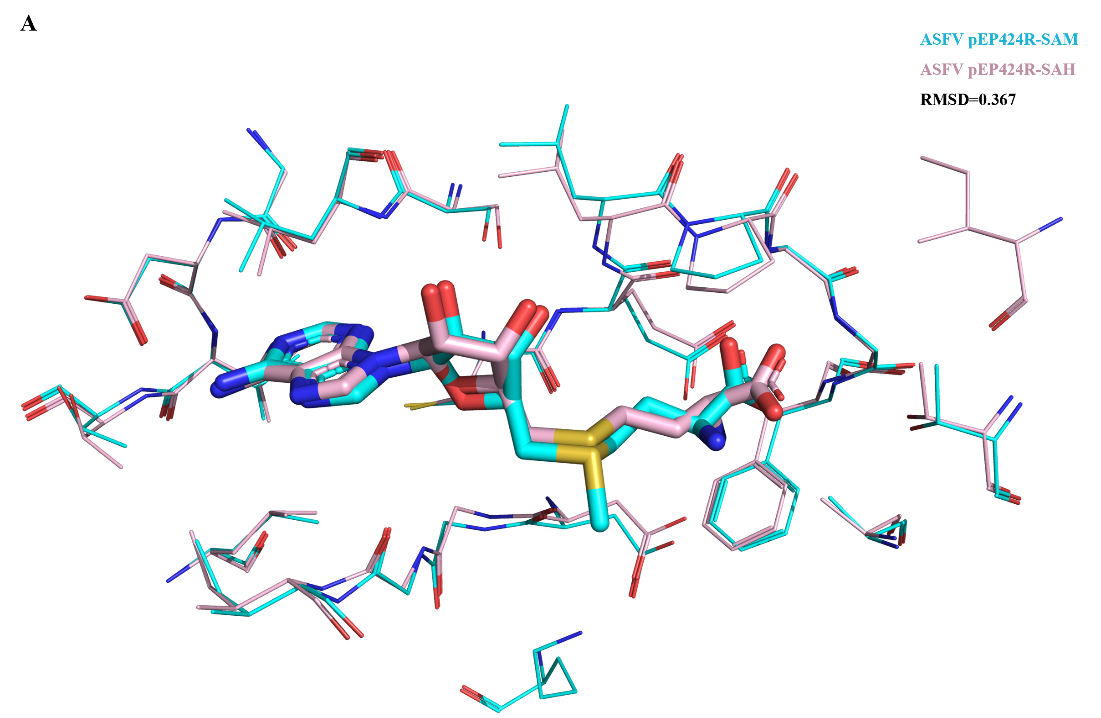


**Figure S4. Comparison of the active pocket before and after pEP424R exerted the enzymatic active function**

(A) Sinefungin, SAH and side chains of selected pEP424R amino acid residues are shown in the stick representation with carbon atoms colored according to the protein/ligand assignment and other elements colored as usual.


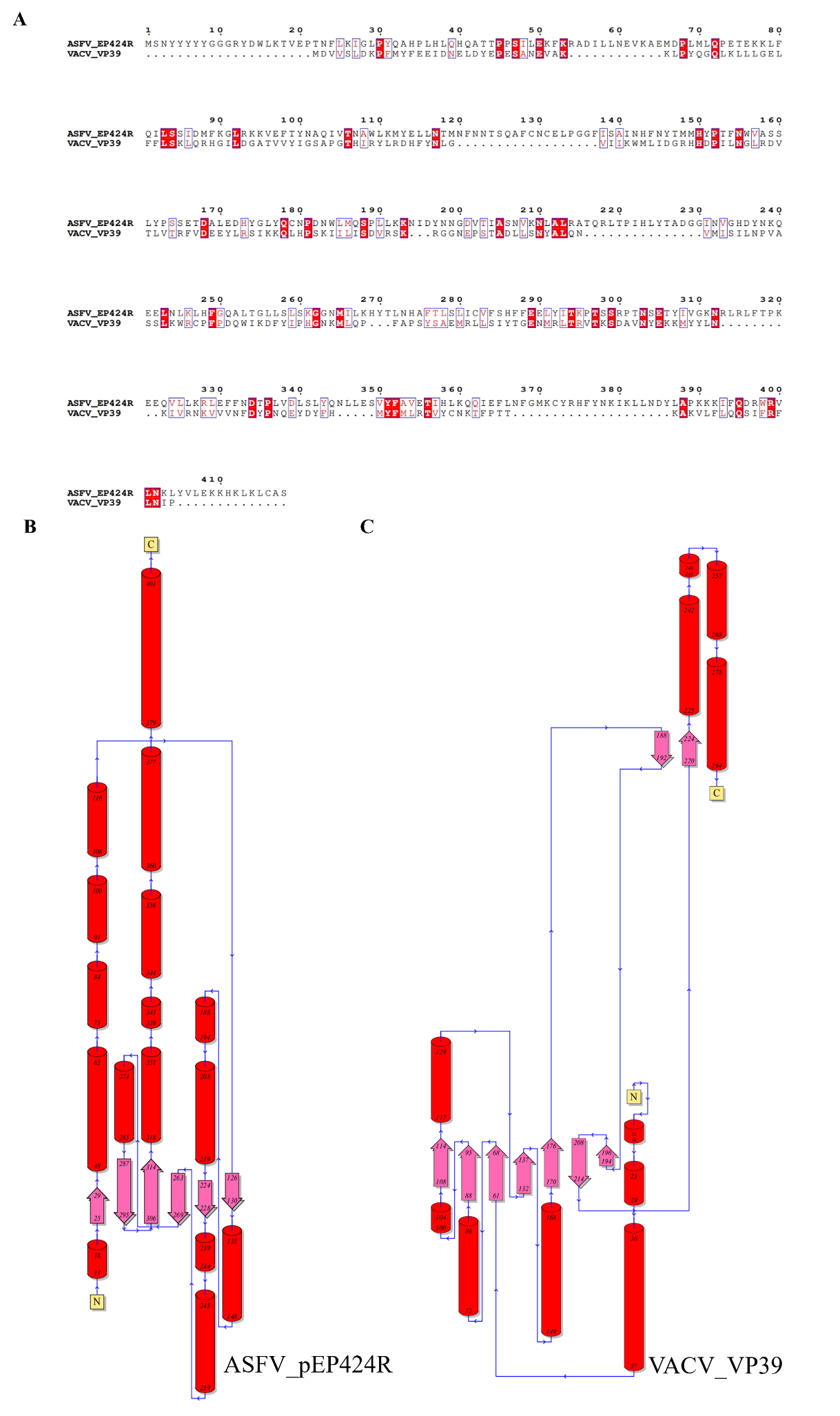


**Figure S5. Overall sequence and structural alignment of ASFV pEP424R and VACV VP39**

(A) Sequence alignment of ASFV pEP424R and VACV VP39. (B) The structural topology diagram of ASFV pEP424R. (C) The structural topology diagram of VACV VP39.


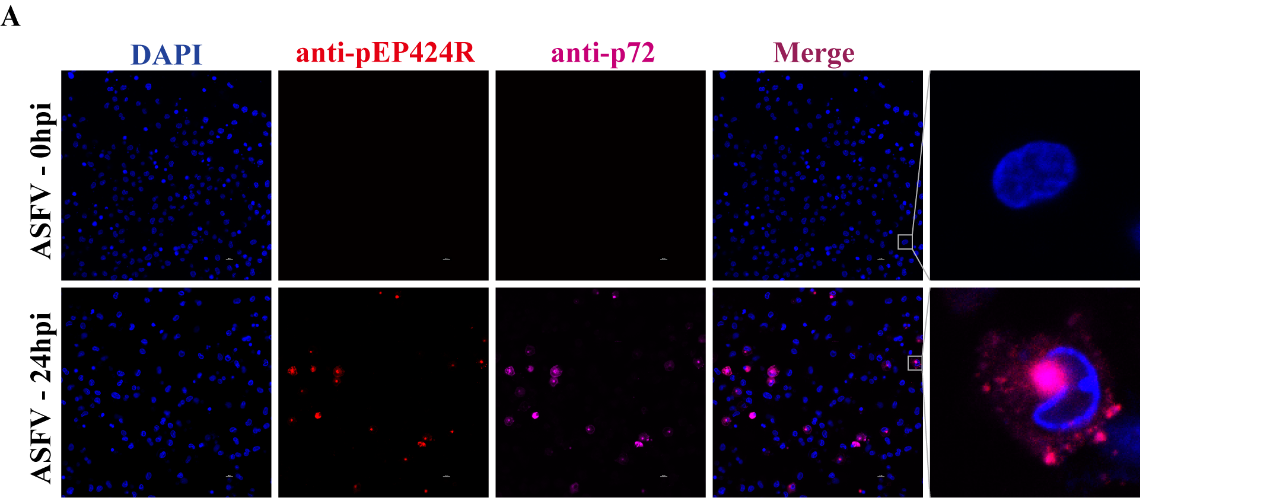


**Figure S6. Analysis of pEP424R colocalization with ASFV VF.**

(A) ASFV-infected PAM cells were fixed at 24 hpi and immunolabeled with homemade mouse anti-pEP424R (red) and rabbit anti-p72 (purple). Bars, 10µm.


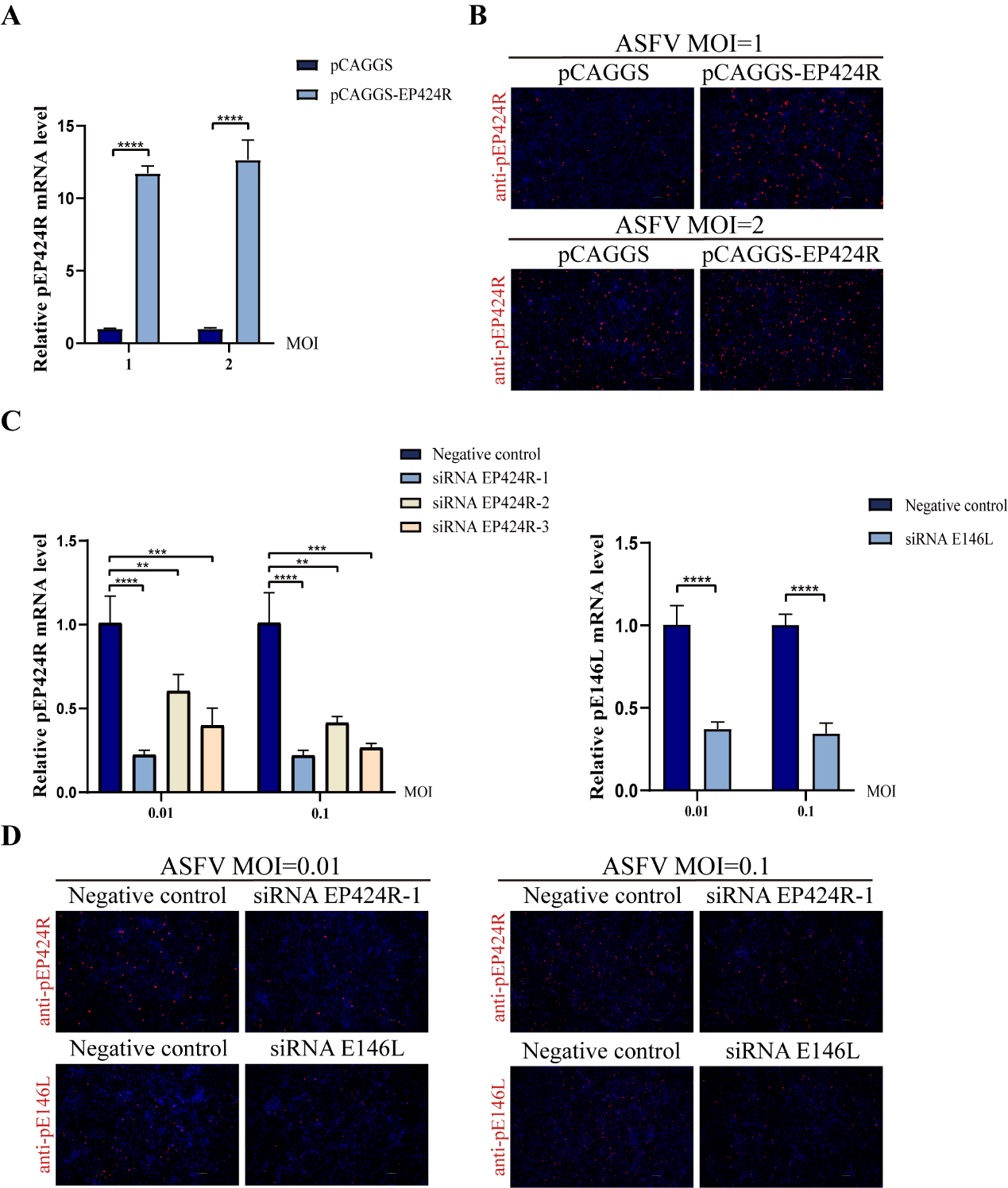


**Figure S7. ASFV pEP424R is crucial for ASFV replication**

(A-B) WSL cells were transfected with empty vector plasmid or pEP424R plasmid. At 24 hours post-transfection, cells were infected with ASFV (MOI = 1 or 2) and collected at 24 hpi to assess the mRNA and protein levels of pEP424R. Scale bar, 100 µm. (C-D) The mRNA and protein level knockdown efficiency of pEP424R and pE146L was assessed under different doses (MOI = 0.01 or 0.1). Scale bar, 100 µm. Data are presented as the mean ± SD of the results from three independent experiments; ns (not significant), * (P<0.05), ** (P<0.01), and *** (P < 0.001).

**Table S1. Primers used in this study**

| **Primer Name** | **Sequence** |
| --- | --- |
| pET42b-EP424R-F | GAAGGAGATATACATATGTCCAATTACTATTAT |
| pET42b-EP424R-R | GGTGGTGGTGGTGGTGGGAGGCACAAAGCTTA |
| pET42b-EP424R-Mut111-F | TATGAGCTGCTAAATACCATGAATTTTAATAATACATCTCAGGC |
| pET42b-EP424R-Mut111-R | ATTTAGCAGCTCATACATAGCAAGCCAAGCAT |
| pET42b-EP424R-Mut228-F | GGGGGATTAAATGTAGGACATGACTACAATAAACAGGAAG |
| pET42b-EP424R-Mut228-R | TCCTACATTAATACCCCCAGCAGCCGTATATAG |
| pET42b-EP424R-Mut268-F | CACTATACCTTAAATCATGCATTTACTCTTTCTTTAATATGTGT |
| pET42b-EP424R-Mut268-R | TGATTTAAGGTATAGTGAGCGAGTATCATGTTTCC |
| pET42b-EP424R-Mut305-F | ACCTATATTGTGGG TAAAAACAGATTACGCTTATTTACCCCC |
| pET42b-EP424R-Mut268-R | TACCCACAATATAGGTAGCAGAGTTTGTGGGC |
| pCAGGS-HA-EP424R-F | GATTACGCTGAATTCATGTCCAATTACTATTATTACTATGGCGG |
| pCAGGS-HA-EP424R-R | AAGATCTGCTAGCTCGAGGAGGGCACAAAGCTTAAG |
| QP-GAPDH-P-F | ACATGGCCTCCAAGGAGTAAGA |
| QP-GAPDH-P-R | GATCGAGTTGGGGCTGTGACT |
| QP-CP204L-F | CGGTAGAATTGTTACGAC |
| QP-CP204L-R | TTCTTGAGCCTGATGTTC |
| QP-B646L-F | CTGCTCATGGTATCAATCTTATCGA |
| QP-B646L-R | GATACCACAAGATCAGGCCGT |
| QP-EP424R-F | TAACGGGGACGTAACCATCG |
| QP-EP424R-R | CCCGTAAGGGCTTGACCAAA |
| QP-E146L-F | GCAAAATCCTCCGAGCTCCT |
| QP- E146L-R | GGGTTGCCTCACAGTTTTCC |

**Table S2. Oligonucleotides for EMSA and BLI**

| **Name** | **Sequence** |
| --- | --- |
| ssRNA (5’-3’) | GACGGGUUAUAUGCAGAGGU |
| dsRNA-sense (5’-3’) | GACGGGUUAUAUGCAGAGGU |
| dsRNA-antisense (5’-3’) | ACCUCUGCAUAUAACCCGUC |
| ssDNA (5’-3’) | GACGGGTTATATGCAGAGGT |
| dsDNA-sense (5’-3’) | GACGGGTTATATGCAGAGGT |
| dsDNA-antisense (5’-3’) | ACCTCTGCATATAACCCGTC |

**Table S3. Small interfering RNAs used in this study**

| **Name** | **Sequence** |
| --- | --- |
| siRNA EP424R-1-sense (5’-3’) | GGACCCACUCAUGUUACAA |
| siRNA EP424R-1-antisense (5’-3’) | UUGUAACAUGAGUGGGUCC |
| siRNA EP424R-2-sense (5’-3’) | GGAAACAGAUGCCCUGGAA |
| siRNA EP424R-2 -antisense (5’-3’) | UUCCAGGGCAUCUGUUUCC |
| siRNA EP424R-3-sense (5’-3’) | GCGGAAACAUGAUACUCAA |
| siRNA EP424R-3 -antisense (5’-3’) | UUGAGUAUCAUGUUUCCGC |
| siRNA-E146L-sense (5’-3’) | CGAUUGACGUAACCGAAGU |
| siRNA-E146L-antisense (5’-3’) | ACUUCGGUUACGUCAAUCG |
